# Supplementary material for: Population-level whole-genome sequencing of Ascochyta rabiei identifies genomic loci associated with isolate aggressiveness
Source: Microb Genom. 2024 Nov 22;10(11):001326. doi: 10.1099/mgen.0.001326 (PMC11893274; doi:10.1099/mgen.0.001326)
Supplement: Uncited Supplementary Material 1. [file mgen-10-01326-s001.pdf]

Population-level whole genome sequencing of *Ascochyta rabiei* identifies genomic loci associated with aggressiveness

Niloofar Vaghefi[1,2], Ido Bar[3*], Jonathan Wanderley Lawley[3], Prabhakaran Thanjavur Sambasivam[3], Melody Christie[3], Rebecca Ford[3]

Author affiliations: [1]Faculty of Science, University of Melbourne, Parkville, Vic 3010, Australia; [2]Centre for Crop Health, University of Southern Queensland, Qld 4350, Australia; [3]Centre for Planetary Health and Food Security, School of Environment and Science, Griffith University, Qld 4111, Australia

*__Correspondence__: i.bar@griffith.edu.au

Keywords: *Ascochyta rabiei*; population genomics; fungal plant pathogen; Ascochyta blight; chickpea; whole genome sequencing

Supp. Table 1. List of *Ascochyta rabiei* isolates collected from 2013 to 2020 in Australia, which were used for whole genome sequencing and variant calling.

Isolate ID	Year	State	Host	Zone	Zone abbreviation	ICC3996	Genesis90	PBA HatTrick	PBA Seamer
FT13092-2	2013	SA	Genesis90	SA Midnorth-Lower Yorke Eyre	SA1	Moderate	High	High	unknown
FT13092-4	2013	SA	Genesis90	SA Midnorth-Lower Yorke Eyre	SA1	Moderate	Moderate	High	unknown
FT13092-6	2013	SA	Genesis90	SA Midnorth-Lower Yorke Eyre	SA1	Low	Moderate	High	unknown
14DON003	2014	VIC	PBA Slasher	SA Vic Mallee	SA/Vic2	Low	Low	High	unknown
TR6400	2014	NSW	PBA HatTrick	NSW NE/Qld SE	NSW/Qld1	Low	Low	High	unknown
TR6408	2014	NSW	PBA HatTrick	NSW NE/Qld SE	NSW/Qld1	Low	Low	Low	unknown
TR6417	2014	NSW	PBA HatTrick	NSW NE/Qld SE	NSW/Qld1	Moderate	High	High	unknown
15CUR001	2015	VIC	Genesis90	SA Vic Mallee	SA/Vic2	Low	Moderate	High	unknown
15CUR002	2015	VIC	Genesis90	SA Vic Mallee	SA/Vic2	Moderate	High	High	unknown
15CUR003	2015	VIC	Genesis90	SA Vic Mallee	SA/Vic2	Low	Low	High	unknown
15DON001	2015	VIC	Genesis90	SA Vic Mallee	SA/Vic2	Low	Low	Low	unknown
15DON007	2015	VIC	PBA Slasher	SA Vic Mallee	SA/Vic2	Moderate	Moderate	High	unknown
F15025	2015	SA	Genesis90	SA Midnorth-Lower Yorke Eyre	SA1	Low	Moderate	High	unknown
F15028	2015	SA	Genesis90	SA Midnorth-Lower Yorke Eyre	SA1	Moderate	Moderate	High	unknown
F15029	2015	SA	Genesis90	SA Midnorth-Lower Yorke Eyre	SA1	Moderate	Moderate	High	unknown
FT15030	2015	SA	Genesis90	SA Midnorth-Lower Yorke Eyre	SA1	Moderate	Moderate	High	unknown
16CUR015	2016	VIC	Genesis90	SA Vic Mallee	SA/Vic2	Low	Moderate	High	Low
16CUR017	2016	VIC	Genesis90	SA Vic Mallee	SA/Vic2	Moderate	Moderate	High	Low
16CUR018	2016	VIC	Genesis90	SA Vic Mallee	SA/Vic2	Moderate	High	High	Moderate
16CUR019	2016	VIC	Genesis90	SA Vic Mallee	SA/Vic2	Moderate	Moderate	High	Low
16RUP012	2016	VIC	Genesis90	SA Vic Bordertown-Wimmera	SA/Vic1	Low	Low	High	Low
16RUP013	2016	VIC	Genesis90	SA Vic Bordertown-Wimmera	SA/Vic1	Low	Low	High	Low
F16083-1	2016	SA	Genesis90	SA Midnorth-Lower Yorke Eyre	SA1	Moderate	Moderate	High	Moderate
F16253-1	2016	SA	Genesis90	SA Midnorth-Lower Yorke Eyre	SA1	Moderate	Moderate	High	Low
TR8102	2016	NSW	PBA HatTrick	NSW NW/Qld SW	NSW/Qld2	Low	Moderate	High	Low

Isolate ID	Year	State	Host	Zone	Zone abbreviation	ICC3996	Genesis90	PBA HatTrick	PBA Seamer
TR8105	2016	NSW	PBA HatTrick	NSW NW/Qld SW	NSW/Qld2	Low	Low	High	Low
17CUR007	2017	VIC	Genesis90	SA Vic Mallee	SA/Vic2	Moderate	Moderate	High	Moderate
F17067-1	2017	SA	Genesis90	SA Vic Bordertown-Wimmera	SA/Vic1	Low	Low	Low	Low
F17076-2	2017	NSW	Genesis90	NSW Central	NSW1	Moderate	High	Moderate	Low
F17175-1	2017	VIC	Genesis90	SA Vic Bordertown-Wimmera	SA/Vic1	Low	Low	Low	Low
F17191-1	2017	SA	Genesis90	SA Midnorth-Lower Yorke Eyre	SA1	High	High	High	High
TR9529	2017	QLD	PBA Seamer	NSW NE/Qld SE	NSW/Qld1	High	High	High	High
TR9538	2017	QLD	PBA Seamer	unknown	unknown	Low	Low	Low	Low
TR9543	2017	QLD	PBA Seamer	unknown	unknown	Low	High	Moderate	Low
TR9544	2017	QLD	PBA Seamer	unknown	unknown	Low	Low	Low	Low
TR9568	2017	NSW	PBA Seamer	NSW NE/Qld SE	NSW/Qld1	Moderate	Moderate	High	Moderate
TR9571	2017	NSW	PBA Seamer	NSW NE/Qld SE	NSW/Qld1	High	High	High	High
AR0002	2020	NSW	PBA Drummond	NSW NE/Qld SE	NSW/Qld1	Moderate	Moderate	Moderate	High
AR0003	2020	NSW	PBA Drummond	NSW NE/Qld SE	NSW/Qld1	Moderate	High	High	Moderate
AR0005	2020	NSW	Kimberly	NSW NE/Qld SE	NSW/Qld1	High	High	High	High
AR0007	2020	NSW	Kimberly	NSW NE/Qld SE	NSW/Qld1	Moderate	Moderate	High	Moderate
AR0009	2020	NSW	ICCU96329	NSW NE/Qld SE	NSW/Qld1	High	High	High	High
AR0013	2020	NSW	ACPFGRIL84	NSW NE/Qld SE	NSW/Qld1	High	High	High	High
AR0015	2020	NSW	ILC05702	NSW NE/Qld SE	NSW/Qld1	High	High	High	High
AR0016	2020	NSW	ICCU96329	NSW NE/Qld SE	NSW/Qld1	High	High	High	High
AR0018	2020	NSW	2-15	NSW NE/Qld SE	NSW/Qld1	High	High	High	High
AR0020	2020	NSW	2-164	NSW NE/Qld SE	NSW/Qld1	High	High	High	High
AR0021	2020	NSW	ILC05658	NSW NE/Qld SE	NSW/Qld1	Moderate	Moderate	High	High
AR0022	2020	NSW	ICCU96329	NSW NE/Qld SE	NSW/Qld1	Moderate	High	Moderate	High
AR0023	2020	NSW	2-216	NSW NE/Qld SE	NSW/Qld1	High	High	High	High
AR0024	2020	NSW	2-15	NSW NE/Qld SE	NSW/Qld1	Moderate	High	High	High
AR0025	2020	NSW	1-131xSel2	NSW NE/Qld SE	NSW/Qld1	Moderate	High	High	High

Isolate ID	Year	State	Host	Zone	Zone abbreviation	ICC3996	Genesis90	PBA HatTrick	PBA Seamer
AR0026	2020	NSW	BG112	NSW NE/Qld SE	NSW/Qld1	Low	Moderate	Moderate	Low
AR0027	2020	NSW	1-131xSel2	NSW NE/Qld SE	NSW/Qld1	High	High	High	High
AR0028	2020	NSW	ICCU96329	NSW NE/Qld SE	NSW/Qld1	Low	Moderate	Low	Moderate
AR0029	2020	NSW	ILC05600	NSW NE/Qld SE	NSW/Qld1	Low	Low	Low	Moderate
AR0031	2020	NSW	unknown	unknown	unknown	Low	High	Moderate	Moderate
AR0032	2020	NSW	CBA 2055	NSW Vic Slopes	NSW/Vic	Low	High	Moderate	Moderate
AR0033	2020	NSW	PBA Slasher	NSW Vic Slopes	NSW/Vic	High	High	High	High
AR0034	2020	NSW	Kyabra	NSW Vic Slopes	NSW/Vic	Low	High	High	Moderate
AR0035	2020	NSW	Genesis90	NSW Vic Slopes	NSW/Vic	Moderate	Low	Moderate	Low
AR0036	2020	NSW	PBA Seamer	NSW Vic Slopes	NSW/Vic	Moderate	Low	Moderate	Low
AR0037	2020	NSW	PBA Boundary	NSW Vic Slopes	NSW/Vic	Low	Low	Low	Moderate
AR0038	2020	NSW	Kyabra	NSW Vic Slopes	NSW/Vic	Low	Moderate	Moderate	Low
AR0039	2020	NSW	PBA Royal	NSW Vic Slopes	NSW/Vic	Low	Low	Low	Moderate
AR0040	2020	NSW	PBA Royal	NSW Vic Slopes	NSW/Vic	Low	Moderate	Moderate	Low
AR0042	2020	NSW	PBA HatTrick	NSW Vic Slopes	NSW/Vic	Moderate	High	Low	Moderate
AR0044	2020	NSW	PBA HatTrick	NSW Vic Slopes	NSW/Vic	Moderate	Moderate	High	Moderate
AR0048	2020	NSW	PBA HatTrick	NSW Vic Slopes	NSW/Vic	Low	High	Low	Moderate
AR0050	2020	NSW	PBA HatTrick	NSW Vic Slopes	NSW/Vic	Low	High	High	Moderate
AR0052	2020	NSW	PBA HatTrick	NSW Vic Slopes	NSW/Vic	Low	Moderate	High	Moderate
AR0055	2020	NSW	PBA HatTrick	NSW Vic Slopes	NSW/Vic	Moderate	High	High	High
AR0056	2020	NSW	PBA HatTrick	NSW Vic Slopes	NSW/Vic	Low	High	High	High
AR0060	2020	NSW	PBA HatTrick	NSW Vic Slopes	NSW/Vic	Low	Low	Low	Low
AR0062	2020	NSW	PBA HatTrick	NSW Vic Slopes	NSW/Vic	Moderate	Low	Moderate	High
AR0063	2020	NSW	PBA HatTrick	NSW Vic Slopes	NSW/Vic	Low	Low	Low	High
AR0064	2020	WA	Amber	WA Sandplain	WA2	High	High	High	Moderate
AR0065	2020	WA	Amber	WA Sandplain	WA2	High	High	High	Moderate
AR0066	2020	WA	Amber	WA Sandplain	WA2	High	High	High	High
AR0067	2020	WA	Amber	WA Sandplain	WA2	Moderate	Moderate	High	High

Isolate ID	Year	State	Host	Zone	Zone abbreviation	ICC3996	Genesis90	PBA HatTrick	PBA Seamer
AR0068	2020	WA	Amber	WA Sandplain	WA2	Moderate	High	High	Moderate
AR0069	2020	WA	Amber	WA Sandplain	WA2	Moderate	High	High	High
AR0070	2020	WA	Amber	WA Sandplain	WA2	Moderate	High	High	High
AR0071	2020	WA	Amber	WA Sandplain	WA2	Moderate	High	High	High
AR0072	2020	WA	Amber	WA Sandplain	WA2	Moderate	High	Moderate	High
AR0073	2020	WA	Amber	WA Sandplain	WA2	Low	Moderate	Low	Low
AR0074	2020	WA	Amber	WA Sandplain	WA2	Low	High	High	High
AR0075	2020	WA	Amber	WA Sandplain	WA2	Moderate	High	High	High
AR0076	2020	WA	Amber	WA Sandplain	WA2	Low	High	High	High
AR0078	2020	WA	Amber	WA Sandplain	WA2	Low	High	High	High
AR0080	2020	WA	Amber	WA Sandplain	WA2	High	High	High	High
AR0081	2020	WA	Amber	WA Sandplain	WA2	Moderate	High	High	High
AR0083	2020	WA	Amber	WA Sandplain	WA2	Moderate	High	High	High
AR0087	2020	WA	Amber	WA Sandplain	WA2	Low	High	High	High
AR0088	2020	NSW	PBA HatTrick	NSW Vic Slopes	NSW/Vic	Moderate	Moderate	High	High
AR0091	2020	NSW	PBA HatTrick	NSW Vic Slopes	NSW/Vic	Low	Low	High	High
AR0093	2020	NSW	PBA HatTrick	NSW Vic Slopes	NSW/Vic	Low	Moderate	Moderate	High
AR0098	2020	NSW	PBA HatTrick	NSW Vic Slopes	NSW/Vic	Low	Low	Low	Moderate
AR0103	2020	NSW	PBA HatTrick	NSW Vic Slopes	NSW/Vic	Low	Low	Moderate	High
AR0105	2020	NSW	CICA1521	NSW NE/Qld SE	NSW/Qld1	Low	Low	Moderate	High
AR0108	2020	NSW	PBA HatTrick	NSW NE/Qld SE	NSW/Qld1	Moderate	Low	High	High
AR0112	2020	QLD	Jimbour	Qld Central	Qld	Low	Low	Low	Low
AR0114	2020	QLD	Jimbour	Qld Central	Qld	Low	Low	Low	Low
AR0115	2020	QLD	Jimbour	Qld Central	Qld	Low	Low	Low	Low
AR0116	2020	QLD	Jimbour	Qld Central	Qld	Low	Low	Low	Low
AR0117	2020	NSW	PBA Seamer	NSW NE/Qld SE	NSW/Qld1	Low	Low	Low	Low
AR0119	2020	NSW	PBA Seamer	NSW NE/Qld SE	NSW/Qld1	Low	Low	Low	Low
AR0122	2020	NSW	PBA Boundary	NSW NE/Qld SE	NSW/Qld1	High	Low	High	High

Isolate ID	Year	State	Host	Zone	Zone abbreviation	ICC3996	Genesis90	PBA HatTrick	PBA Seamer
AR0123	2020	NSW	PBA Boundary	NSW NE/Qld SE	NSW/Qld1	Low	Moderate	High	High
AR0127	2020	NSW	PBA Boundary	NSW NE/Qld SE	NSW/Qld1	Low	Moderate	Moderate	Low
AR0128	2020	NSW	PBA Boundary	NSW NE/Qld SE	NSW/Qld1	High	High	High	High
AR0132	2020	NSW	PBA HatTrick	NSW NE/Qld SE	NSW/Qld1	Moderate	Low	Low	High
AR0133	2020	NSW	PBA HatTrick	NSW NE/Qld SE	NSW/Qld1	Low	Low	Moderate	Moderate
AR0134	2020	NSW	PBA HatTrick	NSW NE/Qld SE	NSW/Qld1	Low	Moderate	Low	High
AR0135	2020	NSW	PBA HatTrick	NSW NE/Qld SE	NSW/Qld1	Low	Moderate	Moderate	Moderate
AR0150	2020	SA	unknown	SA Midnorth-Lower Yorke Eyre	SA1	Moderate	Moderate	High	High
AR0151	2020	SA	unknown	SA Midnorth-Lower Yorke Eyre	SA1	Moderate	Low	Moderate	Moderate
AR0152	2020	SA	unknown	SA Midnorth-Lower Yorke Eyre	SA1	Moderate	Low	Moderate	Moderate
AR0153	2020	SA	Genesis90	SA Midnorth-Lower Yorke Eyre	SA1	Low	Low	Moderate	Moderate
AR0154	2020	SA	Genesis90	SA Midnorth-Lower Yorke Eyre	SA1	Moderate	Low	Moderate	Low
AR0155	2020	SA	Genesis90	SA Midnorth-Lower Yorke Eyre	SA1	Moderate	Low	High	High
AR0156	2020	SA	PBA Monarch	SA Midnorth-Lower Yorke Eyre	SA1	Low	High	Moderate	High
AR0157	2020	SA	PBA Monarch	SA Midnorth-Lower Yorke Eyre	SA1	Moderate	Moderate	Moderate	Moderate
AR0158	2020	SA	PBA Monarch	SA Midnorth-Lower Yorke Eyre	SA1	Moderate	Low	High	Moderate
AR0159	2020	SA	PBA Monarch	SA Midnorth-Lower Yorke Eyre	SA1	Low	Low	Low	Low
AR0160	2020	SA	PBA Monarch	SA Midnorth-Lower Yorke Eyre	SA1	Moderate	Low	Moderate	Moderate
AR0161	2020	SA	PBA Monarch	SA Midnorth-Lower Yorke Eyre	SA1	Low	Low	Low	Low
AR0162	2020	SA	Genesis90	SA Midnorth-Lower Yorke Eyre	SA1	Low	Moderate	High	High
AR0163	2020	SA	Genesis90	SA Midnorth-Lower Yorke Eyre	SA1	Moderate	Low	Moderate	Moderate
AR0164	2020	SA	Genesis90	SA Midnorth-Lower Yorke Eyre	SA1	Low	Moderate	Low	Low
AR0165	2020	SA	PBA Monarch	SA Midnorth-Lower Yorke Eyre	SA1	Low	Low	Moderate	Moderate
AR0166	2020	SA	PBA Monarch	SA Midnorth-Lower Yorke Eyre	SA1	Moderate	Low	High	Moderate
AR0167	2020	SA	PBA Monarch	SA Midnorth-Lower Yorke Eyre	SA1	Moderate	Low	unknown	Low
AR0168	2020	SA	PBA Monarch	SA Midnorth-Lower Yorke Eyre	SA1	Low	Low	Moderate	Moderate
AR0169	2020	SA	PBA Monarch	SA Midnorth-Lower Yorke Eyre	SA1	Moderate	Low	Low	Low
AR0170	2020	SA	PBA Monarch	SA Midnorth-Lower Yorke Eyre	SA1	Moderate	High	High	High

Isolate ID	Year	State	Host	Zone	Zone abbreviation	ICC3996	Genesis90	PBA HatTrick	PBA Seamer
AR0175	2020	WA	Neelam	WA Central	WA1	Moderate	Low	Moderate	Moderate
AR0176	2020	WA	Neelam	WA Central	WA1	Moderate	Low	Low	Low
AR0177	2020	WA	Neelam	WA Central	WA1	Low	Low	Low	Low
AR0178	2020	WA	Neelam	WA Central	WA1	Low	Low	Low	Low
AR0179	2020	WA	Neelam	WA Central	WA1	High	Moderate	Low	Low
AR0180	2020	WA	Neelam	WA Central	WA1	Low	Low	Low	Low
AR0181	2020	WA	Neelam	WA Central	WA1	Low	Low	Low	Moderate
AR0182	2020	WA	Neelam	WA Central	WA1	Low	Low	Low	Low
AR0183	2020	WA	Neelam	WA Central	WA1	Low	Low	Low	Low
AR0184	2020	WA	Neelam	WA Central	WA1	Low	Low	Low	Low
AR0185	2020	WA	Neelam	WA Central	WA1	Low	Moderate	Moderate	Low
AR0186	2020	WA	Neelam	WA Central	WA1	Moderate	Low	Moderate	Low
AR0187	2020	WA	Neelam	WA Central	WA1	Low	Low	Moderate	Low
AR0188	2020	WA	Neelam	WA Central	WA1	Moderate	Low	Moderate	Low
AR0189	2020	WA	Neelam	WA Central	WA1	Low	Low	Low	Low
AR0190	2020	WA	Neelam	WA Central	WA1	Low	Moderate	Moderate	Moderate
AR0191	2020	WA	Neelam	WA Central	WA1	Low	Low	Moderate	Low
AR0192	2020	WA	Neelam	WA Central	WA1	Moderate	High	High	High
AR0193	2020	WA	Neelam	WA Central	WA1	Low	Moderate	Moderate	Low
AR0194	2020	WA	Neelam	WA Central	WA1	Low	Moderate	Low	Low
AR0195	2020	WA	Neelam	WA Central	WA1	Low	Low	Low	Low
AR0196	2020	WA	Neelam	WA Central	WA1	Low	Low	Low	Low
AR0197	2020	WA	Neelam	WA Central	WA1	Moderate	Low	Moderate	Low
AR0198	2020	WA	Neelam	WA Central	WA1	Moderate	Low	Low	Low
AR0199	2020	WA	Neelam	WA Central	WA1	Low	Low	Low	Low
AR0205	2020	NSW	PBA HatTrick	NSW NE/Qld SE	NSW/Qld1	Moderate	Moderate	High	High
AR0206	2020	NSW	PBA HatTrick	NSW NE/Qld SE	NSW/Qld1	Moderate	Moderate	High	High
AR0210	2020	NSW	PBA HatTrick	NSW NW/Qld SW	NSW/Qld2	High	Moderate	High	High

Isolate ID	Year	State	Host	Zone	Zone abbreviation	ICC3996	Genesis90	PBA HatTrick	PBA Seamer
AR0211	2020	NSW	PBA HatTrick	NSW NW/Qld SW	NSW/Qld2	Low	Low	Low	High
AR0212	2020	NSW	PBA HatTrick	NSW NW/Qld SW	NSW/Qld2	Moderate	High	High	High
AR0215	2020	NSW	PBA HatTrick	NSW NW/Qld SW	NSW/Qld2	Low	Low	Moderate	Moderate
AR0216	2020	NSW	PBA HatTrick	NSW Central	NSW1	Low	Moderate	Moderate	High
AR0217	2020	NSW	PBA HatTrick	NSW Central	NSW1	Low	High	Moderate	High
AR0218	2020	NSW	PBA HatTrick	NSW Central	NSW1	Low	Moderate	High	High
AR0219	2020	NSW	PBA HatTrick	NSW Central	NSW1	Low	Low	Low	Low
AR0220	2020	NSW	PBA HatTrick	NSW Central	NSW1	Low	Low	Moderate	Moderate
AR0221	2020	NSW	PBA Seamer	NSW NW/Qld SW	NSW/Qld2	Moderate	Moderate	High	High
AR0222	2020	NSW	PBA Seamer	NSW NW/Qld SW	NSW/Qld2	Low	Low	Low	Moderate
AR0223	2020	NSW	PBA Seamer	NSW NW/Qld SW	NSW/Qld2	Low	High	High	High
AR0225	2020	NSW	PBA Seamer	NSW NW/Qld SW	NSW/Qld2	Low	Low	Moderate	Low
AR0226	2020	NSW	PBA Seamer	NSW NW/Qld SW	NSW/Qld2	Moderate	High	High	High
AR0227	2020	NSW	PBA Seamer	NSW NW/Qld SW	NSW/Qld2	Low	High	Moderate	Low
AR0228	2020	NSW	PBA Seamer	NSW NW/Qld SW	NSW/Qld2	Low	Low	Low	Low
AR0230	2020	NSW	PBA Seamer	NSW NW/Qld SW	NSW/Qld2	Low	Low	Low	Moderate
AR0231	2020	NSW	PBA HatTrick	NSW NW/Qld SW	NSW/Qld2	High	High	High	High
AR0232	2020	NSW	PBA HatTrick	NSW NW/Qld SW	NSW/Qld2	Low	High	High	High
AR0235	2020	NSW	PBA HatTrick	NSW NW/Qld SW	NSW/Qld2	Moderate	High	High	High
AR0236	2020	NSW	PBA HatTrick	NSW NW/Qld SW	NSW/Qld2	Low	High	High	High
AR0237	2020	NSW	PBA HatTrick	NSW NW/Qld SW	NSW/Qld2	Moderate	High	High	High
AR0240	2020	NSW	PBA HatTrick	NSW NW/Qld SW	NSW/Qld2	unknown	unknown	unknown	unknown
AR0241	2020	NSW	PBA HatTrick	NSW NW/Qld SW	NSW/Qld2	Low	Low	High	Moderate
AR0242	2020	NSW	PBA HatTrick	NSW NW/Qld SW	NSW/Qld2	Low	Low	High	Moderate
AR0246	2020	NSW	PBA Seamer	NSW Vic Slopes	NSW/Vic	Moderate	High	High	High
AR0247	2020	NSW	PBA Seamer	NSW Vic Slopes	NSW/Vic	Low	High	High	High
AR0248	2020	NSW	PBA Seamer	NSW Vic Slopes	NSW/Vic	Low	High	High	Moderate
AR0251	2020	NSW	PBA HatTrick	NSW Vic Slopes	NSW/Vic	Moderate	Moderate	High	Moderate

Isolate ID	Year	State	Host	Zone	Zone abbreviation	ICC3996	Genesis90	PBA HatTrick	PBA Seamer
AR0256	2020	NSW	PBA HatTrick	NSW Vic Slopes	NSW/Vic	Moderate	High	High	High
AR0257	2020	NSW	PBA HatTrick	NSW Vic Slopes	NSW/Vic	Low	Low	Moderate	Moderate
AR0261	2020	NSW	unknown	NSW Central	NSW1	Low	Moderate	High	High
AR0262	2020	NSW	unknown	NSW Central	NSW1	Low	Moderate	Moderate	High
AR0263	2020	NSW	unknown	NSW Central	NSW1	Low	Moderate	High	High
AR0264	2020	NSW	unknown	NSW Central	NSW1	Low	Moderate	High	High
AR0265	2020	NSW	unknown	NSW Central	NSW1	Low	Moderate	High	Moderate
AR0266	2020	WA	Amber	WA Sandplain	WA2	Moderate	Moderate	High	High
AR0273	2020	WA	Amber	WA Sandplain	WA2	Moderate	High	High	High
AR0278	2020	NSW	PBA HatTrick	NSW Vic Slopes	NSW/Vic	Low	Moderate	High	High
AR0279	2020	NSW	PBA HatTrick	NSW Vic Slopes	NSW/Vic	Moderate	High	Moderate	Moderate
AR0280	2020	NSW	PBA HatTrick	NSW Vic Slopes	NSW/Vic	Moderate	Moderate	High	High
AR0285	2020	NSW	PBA HatTrick	NSW Vic Slopes	NSW/Vic	Moderate	High	High	High
AR0286	2020	NSW	PBA HatTrick	NSW Vic Slopes	NSW/Vic	Moderate	Moderate	Moderate	Moderate
AR0289	2020	NSW	PBA HatTrick	NSW Vic Slopes	NSW/Vic	Low	High	High	High
AR0290	2020	NSW	PBA HatTrick	NSW Vic Slopes	NSW/Vic	Low	Moderate	High	High
AR0293	2020	VIC	PBA Striker	SA Vic Bordertown-Wimmera	SA/Vic1	Low	Low	High	Moderate
AR0294	2020	VIC	PBA Slasher	SA Vic Bordertown-Wimmera	SA/Vic1	Low	High	Moderate	High
AR0295	2020	VIC	PBA Royal	SA Vic Bordertown-Wimmera	SA/Vic1	Low	Moderate	Moderate	Moderate
AR0296	2020	VIC	Kalkee	SA Vic Bordertown-Wimmera	SA/Vic1	Low	Low	High	High
AR0297	2020	VIC	PBA Monarch	SA Vic Bordertown-Wimmera	SA/Vic1	Low	High	High	High
AR0298	2020	VIC	Genesis90	SA Vic Bordertown-Wimmera	SA/Vic1	Moderate	Low	High	High
AR0299	2020	SA	PBA Striker	SA Vic Mallee	SA/Vic2	High	Moderate	High	Low
AR0300	2020	VIC	PBA Striker	SA Vic Bordertown-Wimmera	SA/Vic1	Moderate	High	High	High
AR0301	2020	VIC	Genesis90	SA Vic Bordertown-Wimmera	SA/Vic1	Moderate	High	High	High
AR0302	2020	VIC	Genesis90	SA Vic Mallee	SA/Vic2	Low	Low	High	High
AR0303	2020	VIC	Genesis90	SA Vic Mallee	SA/Vic2	Low	High	High	Low
AR0304	2020	VIC	Genesis90	SA Vic Bordertown-Wimmera	SA/Vic1	Moderate	High	High	Moderate

Isolate ID	Year	State	Host	Zone	Zone abbreviation	ICC3996	Genesis90	PBA HatTrick	PBA Seamer
AR0312	2020	WA	PBA Striker	WA Central	WA1	Low	Moderate	Moderate	Moderate
AR0313	2020	WA	PBA Striker	WA Central	WA1	Low	Moderate	Moderate	Moderate
AR0314	2020	WA	PBA Striker	WA Central	WA1	Moderate	High	Moderate	Low
AR0315	2020	WA	PBA Striker	WA Central	WA1	Moderate	High	High	High
AR0316	2020	WA	PBA Striker	WA Central	WA1	Low	Moderate	High	High
AR0317	2020	WA	PBA Striker	WA Central	WA1	Low	Low	High	High
AR0318	2020	WA	PBA Striker	WA Central	WA1	High	High	High	High
AR0319	2020	WA	PBA Striker	WA Central	WA1	Moderate	Low	High	Moderate
AR0320	2020	WA	PBA Striker	WA Central	WA1	Moderate	High	High	High
AR0321	2020	WA	PBA Striker	WA Central	WA1	Moderate	High	High	High

Isolate information was retrieved from the *Ascochyta dashboard* on June 30th 2022.

Supp. Table 2. Summary of analysis of molecular variance (AMOVA) results for 230 *Ascochyta rabiei* isolates collected in 2013, 2014, 2015, 2016, 2017, and 2020 from different States and locations in Australia.

Source	d.f.[a]	SS[b]	MS[c]	variance	proportion of variation	*P* value
**Among years**	5	2,398.63	479.72	2.37	1.0%	0.322
**Among States within years**	11	7,7904.73	718.61	30.34	13.0%	0.001
**Among locations within States**	34	12,111.780	367.02	46.94	21.0%	0.001
**Within samples**	179	25,472.79	143.10	143.11	65.0%	0.001
**Total**	229	47,887.94	210.96	222.76	100.0%	-

[a]Degree of freedom
[b]Sum of squared observations
[c]Mean of squared observations

Supp. Table 3. Summary of analysis of molecular variance (AMOVA) results for 230 *Ascochyta rabiei* isolates collected in 2013, 2014, 2015, 2016, 2017, and 2020 from different agroecological zones and locations in Australia.

Source	d.f.[a]	SS[b]	MS[c]	variance	proportion of variation	*P* value
**Among years**	5	2,398.63	382.84	7.34	3.0%	0.143
**Among zones within years**	20	13,367.88	601.58	40.43	17.5%	0.001
**Among locations within Zone**	25	6,648.63	235.76	30.59	14.0%	0.001
**Within samples**	179	25,472.80	127.66	143.11	65.5%	0.001
**Total**	229	47,887.94	186.42	221.47	100.0%	-

[a]Degree of freedom
[b]Sum of squared observations
[c]Mean of squared observations

Supp. Table 4. Indices of genetic diversity of the Australian *Ascochyta rabiei* populations defined as groups of isolates collected from agroecological zones.

Population[a]	N[b]	MLL[c]	eMLL[d]	SE[e]	λ[f]	E5[g]	CF[h]	Hexp[i]
NSW1	11	10	9.18	3.86e-01	0.98	0.95	9%	0.00
NSW/Qld1	39	32	9.48	6.67e-01	0.98	0.89	17%	0.00
NSW/Qld2	22	14	8.09	9.65e-01	0.95	0.88	36%	0.00
NSW/Vic	37	26	9.05	8.41e-01	0.97	0.88	30%	0.00
SA1	31	29	9.81	4.12e-01	0.99	0.96	6%	0.00
SA/Vic1	13	13	-	-	-	-	-	-
SA/Vic2	14	14	-	-	-	-	-	-
Qld	4	4	-	-	-	-	-	-
WA1	35	23	8.69	9.58e-01	0.96	0.82	34%	0.00
WA2	20	18	9.53	5.80e-01	0.95	0.95	10%	0.00

[a] Agroecological zones include NSW1: NSW Central, NSW/Qld1: NSW NE/Qld SE, NSW/Qld2: NSW NW/Qld SW, NSW/Vic: NSW Victoria slopes, Qld: Queensland Central, SA1: SA Midnorth-Lower Yorke Eyre, SA/Vic1: SA Vic Bordertown-Wimmera, SA/Vic2: SA Vic Mallee, WA1: WA Central, WA2: WA Sandplain (Fig. 1).

[b] N = population size.

[c] MLL = Number of multi-locus lineages after contracting the data set using the *cutoff_predictor* and *mlg.filter* functions in *poppr* (1,2).

[d] eMLL = expected number of MLLs at the lowest common sample size to allow comparison between populations.

[e] SE = The standard error for the rarefaction analysis for eMLLs.

[f] λ = defined here as unbiased Simpson's complement index of genotypic diversity, defined as the probability that two genotypes randomly chosen from the population are different (3), corrected for sample size.

[g] E5 = Evenness as a measure of the distribution of MLL abundances, where in a population with equally abundant MLLs, E5 = 1, and in a population dominated by a single MLL, E5=0.

[h] CF = Clonal fraction; (N-number of MLLs)/N, where N is total number of isolates.

[i] H_{exp} = Nei's gene diversity (expected heterozygosity) (4).

Supp. Table 5. Genetic variations with significant impact on aggressiveness of *Ascochyta rabiei* on different chickpea cultivars based on pyseer (5) results. Variant type and impact based on SNPEff (6) and putative function of the adjacent loci based on BLASTp results are given. Predector score is provided with EffectorP 3.0 score in parentheses. Non-effector indicates a negative Predector score and > 0.5 non-effector score from EffectorP 3.0**.**

**Variant**	**Variation also detected in DAPC**	**DNA region detected in DAPC**	***p* value**				**Variant type and impact**	**Putative protein function**	**Predector Score (EffectorP 3.0 score)**	**Repeat family**
			**Genesis 090**	**ICC3996**	**PBA HatTrick**	**PBA Seamer**				
ctg01_1654657	Yes	Yes, region 3	-	-	-	0.0273	intergenic_region, modifier, EKO05_000484-EKO05_000485	EKO05_000484: hypothetical protein with high similarity to fungal GTPases, EKO05_000485 hypothetical DNA binding protein with a transmembrane domain	non-effectors	LTR/Copia
ctg01_1665154	Yes	Yes, region 3	-	-	-	0.0273	same as ctg01_1654657			
ctg01_2020023	-	-	-	-	-	0.0321	synonymous_variant, low impact, EKO05_000605	hypothetical protein of unknown function with similarity to fungal peptidases	-3.346 (0.716: Cytoplasmic effector but no signal peptide detected)	-
ctg01_3303380	-	-		-	-	0.0321	intergenic_region, modifier, EKO05_001003-EKO05_001004	EKO05_001003: **hypothetical apoplastic effector**, EKO05_001004: hypothetical protein of unknown function	EKO05_001003: 1.475 (0.808: Apoplastic effector), EKO05_001004: non-effector	LTR/Gypsy
ctg02_2690139	Yes	Yes, region 8	-	-	-	0.0273	missense_variant, moderate impact, EKO05_001768	hypothetical protein with similarity to fungal transcription factors with a zinc-finger domain	non-effector	-
ctg02_325563	-	Yes, region 6	-	-	-	0.0347	intergenic_region, modifier, EKO05_001071-EKO05_001072	EKO05_001071: hypothetical HET domain containing protein with transmembrane domain, EKO05_001072: Phosphotransferase	non-effectors	LTR/Gypsy
ctg02_364191	Yes	Yes, region 6	-	-	-	0.0273	same as ctg_02_325563			

ctg02_83011	-	-	-	-	-	0.0347	intergenic_region, modifier, EKO05_001011-EKO05_001012	EKO05_001011**: hypothetical Apoplastic/cytoplasmic effector**, EKO05_001012: **hypothetical effector with similarity to pep1 protein**	EKO05_001011: 1.873 (0.832, Apoplastic /cytoplasmic effector, signal peptide detected), EKO05_001012: 0.841 (0.579, Apoplastic effector, signal peptide detected)	DNA/TcMar -Fot1
ctg03_1415178	Yes	Yes, region 12	-	-	-	0.0273	intergenic_region, modifier, EKO05_002061-EKO05_002062	EKO05_002061: hypothetical protein of unknown function, EKO05_002062: **hypothetical apoplastic effector**	EKO05_002061: non-effector, EKO05_002062: 0.676 (0.709, Apoplastic effector)	LTR/Gypsy
ctg03_1428702	-	Yes, region 12	-	-	-	0.0347	same as ctg_03_1415178			
ctg03_2025011	-	Yes, region 14	-	-	0.0036	0.0339	intergenic_region, modifier, EKO05_002252-EKO05_002253	EKO05_002252-EKO05: hypothetical protein of unknown function, EKO05_002253: hypothetical protein with similarity to oxidoreductases	non-effectors	LTR/Gypsy
ctg03_357028	-	-	-	-	0.0489	0.0143	intergenic_region, modifier, EKO05_001820, EKO05_001821	EKO05_001820: hypothetical protein of unknown function with a transmembrane domain, EKO05_001821: hypothetical protein of unknown function	non-effectors	LTR/Copia
ctg03_479997	-	-	-	-	-	0.0321	intergenic_region, modifier, EKO05_001827-EKO05_001828	EKO05_001827: hypothetical protein with similarity to fungal Salicylate monooxygenases, EKO05_001828: **hypothetical apoplastic effector**	EKO05_001827: non-effector, EKO05_001828: 1.578 (0.861 Apoplastic effector)	LTR/Gypsy
ctg04_2574966	-	-	-	-	0.0185	-	intergenic_region, modifier, EKO05_003200	hypothetical protein of unknown function	non-effector	DNA/hAT-Charlie
ctg05_77541	-	-	-	-	-	0.0347	intergenic_region, modifier, EKO05_003204-EKO05_003205	hypothetical proteins of unknown function	non-effectors	LTR/Gypsy

ctg07_1292465	-	-	-	-	-	0.0361	missense_variant, moderate impact, EKO05_005029	hypothetical alkaline phosphatase	0.561 (non-effector), signal peptide detected	-
ctg07_899731	-	-	-	-	-	0.0361	missense_variant, moderate impact, EKO05_004868	hypothetical protein of unknown function	non-effector	-
ctg08_1872808	-	Yes, region 31	-	-	0.0109	-	intergenic_region, modifier, EKO05_005581-CHR_END	hypothetical protein with transmembrane domains and high similarity to hexose transporters in fungi	non-effector	LTR/Gypsy
ctg08_1872842	-	Yes, region 31	0.02	-	0.0102	-	same as ctg_08_1872808			
ctg08_1872963	-	Yes, region 31	0.0182	0.0165	0.0000188	0.00167	same as ctg_08_1872808			
ctg08_1875370	-	Yes, region 31	-	-	0.0102	-	same as ctg_08_1872808			
ctg08_1875396	-	Yes, region 31	-	-	0.0437	-	same as ctg_08_1872808			
ctg08_1875471	-	Yes, region 31	-	-	0.00528	-	same as ctg_08_1872808			
ctg08_448981	Yes	Yes, region 28	-	-	-	0.0364	intergenic_region, modifier, EKO05_005280-EKO05_005281	EKO05_005280-EKO05: **hypothetical apoplastic effector**, EKO05_005281: hypothetical protein of unknown function	EKO05_005280: -2.911 (0.509, Apoplastic effector), EKO05_005281: non-effector	LTR/Gypsy
ctg08_98214	Yes	Yes, region 27	-	-	-	0.0273	intergenic_region, modifier, EKO05_005201-EKO05_005202	EKO05_005201: hypothetical apoplastic effector, EKO05_005202: hypothetical protein of unknown function	EKO05_005201: -1.476 (0.654, Apoplastic effector), EKO05_005202: -3.378 (0.675 Cytoplasmic effector but no signal peptide detected)	LTR/Gypsy

ctg09_169049	-	-	-	-	-	0.0347	upstream_gene_variant, modifier, EKO05_005613, intergenic_region, modifier, EKO05_005611-EKO05_005612	EKO05_005611: hypothetical protein with similarity to fungal polysaccharide lyases, EKO05_005612: hypothetical RNA methyl-transferase, EKO05_005613: hypothetical protein with similarity to fungal transcription factors with a zinc-finger domain	EKO05_005611: 0.688 (non-effector), signal peptide detected, EKO05_005612: -2.433 (0.577, Cytoplasmic effector, no signal peptide detected), EKO05_005613: non-effector	LTR/Gypsy
ctg09_1747152	-	-	-	-	-	0.0347	intergenic_region, modifier, EKO05_006084-EKO05_006085	EKO05_006084: hypothetical protein of unknown function, EKO05_006085: hypothetical aspartate ammonia-lyase	non-effectors	LTR/Gypsy
ctg09_54897	-	-	-	0.0305	-	-	intergenic_region, modifier, EKO05_005587-EKO05_005588	EKO05_00558: hypothetical DNA helicase, EKO05_005588: hypothetical protein with similarity to 2-dehydropantoate 2-reductase	non-effectors	unknown repeat family
ctg10_10643	Yes	Yes, region 34	-	-	0.00895	0.0374	synonymous_variant, low impact, EKO05_006140	hypothetical protein of unknown function	-2.614 (0.522, Cytoplasmic effector but no signal peptide detected)	DNA/Kolobok-H
ctg10_157955	Yes	Yes, region 35	-	-	-	0.0364	intergenic_region, modifier, EKO05_006148-EKO05_006149	EKO05_006148: hypothtical protein of unknown function, EKO05_006149: hypothetical oxidooreductase	non-effectors	DNA/TcMar-Fot1
ctg10_157964	Yes	Yes, region 35	-	-	-	0.0438	Same as ctg_10_157955			
ctg10_158053	Yes	Yes, region 35	-	-	-	0.0364	Same as ctg_10_157955			
ctg10_1866672	-	-	-	-	-	0.0347	intergenic_region, modifier, EKO05_006715-EKO05_006717	EKO05_006715: hypothetical protein of unknown function with transmembrane domains, EKO05_006717	EKO05_006715: non-effector, EKO05_006717: -2.708 (0.627, Cytoplasmic effector but no sugnal peptide detected)	-

ctg12_1225837	-	Yes, region 45	-	0.0126	-	-	intergenic_region, modifier, EKO05_007471-EKO05_007472	EKO05_007471: hypothetical protein of unknown function, EKO05_007472: **hypothetical cytoplasmic effector**	EKO05_007471: non-effector, EKO05_007472: -1.071 (0.535, Cytoplasmic effector, signal peptide detected)	LTR/Gypsy
ctg12_1234964	Yes	Yes, region 45	-	-	-	0.0273	same as ctg_12_1225837			
ctg12_1234984	Yes	Yes, region 45	-	-	-	0.0273	same as ctg_12_1225837			
ctg12_1530432	-	-	-	-	-	0.00737	intergenic_region, modifier, EKO05_007577-EKO05_007578	EKO05_007577: hypothetical HECT-type E3 ubiquitin transferase, EKO05_007578: hypothetical cytoplasmic protein of unknown function	EKO05_007577: non-effector, EKO05_007578: 0.811 (non-effector)	-
ctg12_763572	-	-	-	-	0.0489	0.0143	upstream_gene_variant, modifier, EKO05_007339, downstream_gene_variant, modifier, EKO05_007336, EKO05_007337, EKO05_007338	EKO05_007336: hypothetical protein with similarity to MYG1 and fungal protein hydrolases, EKO05_007337, EKO05_007338: hypothetical GCD complex subunit gcd7, EKO05_007339: hypothetical protein-serine/threonine phosphatase	EKO05_007336: -3.188 (0.,628, Cytoplasmic effector but no signal peptide detected), EKO05_007337: -2.372 (0.732, Cytoplasmic effector but no signal peptide detected), EKO05_007338: non-effector, EKO05_007339: non-effector	-
ctg12_763578	-	-	-	-	0.0489	0.0143	same as ctg_12_763572			-
ctg12_763589	-	-	-	-	0.0489	0.0143	same as ctg_12_763572			-

ctg13_1358937	-	Yes, region 50	0.0275	0.0225	-	-	intergenic_region, modifier, EKO05_007941-EKO05_007942	EKO05_007941: hypothetical protein with transmembrane domain siliar to fungal oligopeptide transporters, EKO05_007942: hypothetical protein of unknown function	non-effectors	LTR/Gypsy
ctg13_1474524	-	Yes, region 51	-	-	-	0.0347	intergenic_region, modifier, EKO05_007958-EKO05_007959	EKO05_007958: hypothetical glycoside hydrolase, EKO05_007959: hypothetical protein of unknown function	EKO05_007958: -0.94 (0.878, Apoplastic effector), EKO05_007959: non-effector	DNA/TcMar -Fot1
ctg13_192218	-	-	-	-	-	0.0347	intergenic_region, modifier, EKO05_007660-EKO05_007661	EKO05_007660: PQ-Loop domain containing protein, EKO05_007661: hypothetical protein with similarity to fungal DNA polymerases	non-effectors	DNA/Merlin
ctg13_842542	-	-	-	-	-	0.0347	intergenic_region, modifier, EKO05_007845-EKO05_007846	EKO05_007845: hypothetical protein of unknown function with transmembrane domains, EKO05_007846: hypothetical protein with similarity to clavaminate synthase proteins in fungi	EKO05_007845: non-effector, EKO05_007846: non-effector	LTR/Gypsy
ctg13_842613	-	-	-	-	-	0.0347	same as ctg_13_842542			
ctg14_1472702	-	Yes, region 55	-	0.0269	-	-	intergenic_region, modifier, EKO05_008461-EKO05_008462	EKO05_008461 hypothetical transmembrane protein, putative Mg transporter zinc transport protein, EKO05_008462: hypothetical apoplastic effector	EKO05_008461: non effector, EKO05_008462: 0.847 (0.671 - Apoplastic effector)	LTR/Gypsy
ctg14_1472722	-	Yes, region 55	-	0.014	-	-	same as ctg_14_1472702			
ctg14_1472755	-	Yes, region 55	-	0.0156	-	-	same as ctg_14_1472702			
ctg15_1247671	-	-	-	-	-	0.0347	intergenic_region, modifier, EKO05_008834-EKO05_008835	EKO05_008834: hypothetical transcription factor, EKO05_008835: Hypothetical protein with similarity to glycerate kinanses in fungi	non-effectors	LTR/Copia

ctg15_1503691	-	Yes, region 62	0.0179	-	0.0154	-	downstream_gene_variant, modifier, EKO05_008905	EKO05_008905: DNA helicase with a transmembrane domain	non-effectors	unknown family
ctg15_1503696	Yes	Yes, region 62	0.0199	-	-	-	same as ctg_15_1503691			
ctg15_1503844	-	Yes, region 62	0.00481	0.0155	0.00797	-	same as ctg_15_1503691			
ctg15_1504039	-	Yes, region 62	0.00481	0.0155	0.00797	-	same as ctg_15_1503691			
ctg15_1504127	-	Yes, region 62	0.00481	0.0155	0.00797	-	same as ctg_15_1503691			
ctg15_1504334	-	Yes, region 62	0.0166	0.0131	0.0236	-	same as ctg_15_1503691			
ctg17_452611	-	-	-	-	-	0.0347	intergenic_region, modifier, EKO05_009528-EKO05_009529: hypothetical vitamin B6 transporter	EKO05_009528: hypothetical protein disulfide-isomerase, EKO05_009529: non-effector	EKO05_009528: 0.254 (0.511, Cytoplasmic effector, signal peptide detected), EKO05_009529	
ctg19_104632	-	-	-	-	0.0489	0.0143	downstream_gene_variant, modifier, EKO05_010260	hypothetical protein of unknown function	non-effector	DNA/TcMar-Fot1
ctg19_1102753	-	-	-	-	0.0146	0.019	intergenic_region, modifier, EKO05_010490-EKO05_010491	EKO05_010490: hypothetical acetylxylan esterase, EKO05_010491: **hypothetical apoplastic effector**	EKO05_010490: 0.82 (non-effector), EKO05_010491: 2.117 (0.835: Apoplastic effector), signal peptide detected	LTR/Gypsy
ctg19_1102770	-	-	-	-	0.0421	0.0116	same as ctg_19_1102753			
ctg19_1102779	-	-	-	-	0.0421	0.0116	same as ctg_19_1102753			

ctg19_1154999	yes	Yes, region 71	0.00638	-	0.00293	0.000122	intergenic_region, modifier, EKO05_010492-EKO05_010493	EKO05_010492: hypothetical apoplastic effector, EKO05_010493: amino acid transporter with transmembrane domains	EKO05_010492: 2.244 (0.895, Apoplastic effector), EKO05_010493: non-effector	LTR/Gypsy
ctg19_1155004	yes	Yes, region 71	0.00638	-	0.00293	0.000122	same as ctg_19_1154999			
ctg19_1294610	-	-	-	-	-	0.0191	intergenic_region, modifier, EKO05_010512-EKO05_010513	EKO05_010512: hypothetical protein with a transmembrane domain; EKO05_010513: hypothetical protein, putative metal ion binding	EKO05_010512: -598 (0.636, Cytoplamic effector but no signal peptide detected), EKO05_010513: non-effector	DNA/TcMar-Fot1
ctg19_422984	-	-	-	-	-	0.0347	downstream_gene_variant, modifier, EKO05_010323, intergenic_region, modifier, EKO05_010324-EKO05_010325	EKO05_010323: hypothetical apoplastic effect, EKO05_010324: hypothetical protein of unknown function, EKO05_010325: dehydroquinate dehydratase	EKO05_010323: -0.15 (0.560, Apoplastic protein), EKO05_010324: non-effector, EKO05_010325: non-effector	LTR/Gypsy
ctg20_20369	-	-	-	-	0.0269	0.0394	upstream_gene_variant, modifier, EKO05_010525, intergenic_region, modifier, EKO05_010525-EKO05_010526	EKO05_010525: hypothetical protein of unknown function with a transmembrane domain, EKO05_010526: hypothetical protein of unknown function	EKO05_010525: -3.346 (0.608, cytoplasmic effector but no signal peptide detected), EKO05_010526: non-effector	-
ctg20_20645	-	-	-	-	0.0378	0.0124	same as ctg_20_20369			
ctg21_487980	-	-	-	-	-	0.0347	missense_variant, moderate impact, EKO05_010986	hypothetical protein of unknown function	non-effector	-

Supp. Table 6. Genetic variations predicted to be associated with aggressiveness of Ascochyta rabiei isolates on different chickpea cultivars detected using DAPC analyses. Loading refers to the contribution of each locus to the observed differentiation, and sub-population refers to the clonal lineage (subpopulation) of isolates within which the association was detected (clusters/subpopulations detected in Figure 1). Predector score is provided with EffectorP 3.0 score in parentheses. Non-effector indicates a negative Predector score and > 0.5 non-effector score from EffectorP 3.0.

Region	Variation	Chickpea variety	Loading	Sub-population	Variant type and location	Putative protein function	Predector Score (EffectorP 3.0 score)	Repeat family	region detected in Pyseer
1	ctg01_847649	Genesis090	0.00519917	Cluster3	synonymous_variant, low impact, EKO05_000231	Hypothetical protein with similarity to fungal RNA helicases	non-effector	-	No
	ctg01_847688	Genesis090	0.00639330	Cluster3	same as ctg01_847649				
2	ctg01_1510437	PBA HatTrick	0.00638156	Cluster2	missense_variant, moderate impact, EKO05_000430	hypothetical protein of unknown function	non-effector	-	No
3	ctg01_1654657	PBA Seamer	0.01106706	Cluster 1	intergenic_region, modifier, EKO05_000484-EKO05_000485	EKO05_000484: hypothetical protein with high similarity to fungal GTPases, EKO05_000485 hypothetical DNA binding protein with a transmembrane protein	non-effectors	LTR/Copia	Yes
	ctg01_1665154	PBA Seamer	0.01106706	Cluster 1	same as ctg01_1654657				
4	ctg01_3363573	Genesis090	0.01889603	Cluster 2	synonymous_variant, low impact, EKO05_001005	hypothetical protein of unknown function	non-effector	DNA/Kolobok-H	No
		ICC3996	0.00533564	Cluster 2					
5	ctg02_5952	PBA HatTrick	0.00670503	Cluster 1	upstream_gene_variant, modifier, EKO05_001006	hypothetical protein of unknown function	-2.078 (0.519, cytoplasmic effector but no signal peptide detected)	DNA/Kolobok-H	No
	ctg02_7704	ICC3996	0.00964527	Cluster 1	same as ctg02_5952				
		PBA HatTrick	0.00800386	Cluster 1					

6	ctg02_352849	PBA Seamer	0.01106706	Cluster 1	intergenic_region, modifier, EKO05_001071-EKO05_001072	EKO05_001071: hypothetical HET domain containing protein with a transmembrane domain, EKO05_001072: Phosphotransferase	non-effectors	LTR/Gypsy	Yes
	ctg02_364191	PBA Seamer	0.01106706	Cluster 1	same as ctg02_352849				
7	ctg02_1660285	PBA Seamer	0.00772822	Cluster 2	missense_variant, moderate impact, EKO05_001443	Hypothetical protein with similarity to fungal RNA helicases	non-effector	-	No
8	ctg02_2690139	PBA Seamer	0.01106706	Cluster 1	missense_variant, moderate impact, EKO05_001768	hypothetical protein with similarity to fungal transcription factors with a zinc-finger domain	non-effector	-	Yes
9	ctg02_2788591	PBA HatTrick	0.00638157	Cluster 2	intergenic_region, modifier, EKO05_001778-EKO05_001779	EKO05_001778: Hypothetical RNA helicase, EKO05_001779: hypothetical protein of unknown function	EKO05_001778: non-effector, EKO05_001779: 3.941 (0.862, Apoplastic effector)	LTR	No
10	ctg02_2843207	ICC3996	0.01253206	Cluster 1	intergenic_region, modifier, EKO05_001780-CHR_END	hypothetical protein of unknown function	-2.613 (0.567, Cytoplasmic effector but no signal peptide detected)	DNA/Kolobok-H	No
11	ctg03_401008	PBA HatTrick	0.00638157	Cluster 2	intragenic_variant, modifier, EKO05_001821-EKO05_001822	Hypothetical proteins of unknown function	EKO05_001821: non-effector, EKO05_001822: -2.643 (0.846, Cytoplasmic effector but no signal peptide)	LTR	No

12	ctg03_1415178	PBA Seamer	0.01106706	Cluster 1	intergenic_region, modifier, EKO05_002061-EKO05_002062	EKO05_002061: hypothetical protein of unknown function, EKO05_002062: **hypothetical apoplastic effector**	EKO05_002061: non-effector, EKO05_002062: 0.676 (0.709, Apoplastic effector)	LTR/Gypsy	Yes
13	ctg03_1878424	PBA Seamer	0.00650038	Cluster 1	missense_variant, moderate impact, EKO05_002214	Hypothetical protein of unknown functions	non-effector	-	No
14	ctg03_2014060	PBA HatTrick	0.00638156	Cluster 2	intergenic_region, modifier, EKO05_002252-EKO05_002253	EKO05_002252-EKO05: hypothetical protein of unknown function, EKO05_002253: hypothetical protein with similarity to oxidoreductases	non-effectors	LTR/Gypsy	No
	ctg03_2025024	Genesis090	0.03814387	Cluster 2	same as ctg03_2014060				
	ctg03_2039741	PBA Seamer	0.00772822	Cluster 2	same as ctg03_2014060				
15	ctg03_2550442	PBA HatTrick	0.00638157	Cluster 2	intergenic_region, modifier, EKO05_002440-EKO05_002441	EKO05_002440: hypothetical protein of unknown function, EKO05_002441: hypothetical protein with similarity to fungal Salicylate monooxygenases	non-effectors	LTR/Gypsy	No
	ctg03_2550592	PBA HatTrick	0.00638157	Cluster 2	same as ctg03_2550442				
	ctg03_2556946	PBA HatTrick	0.00638157	Cluster 2	same as ctg03_2550442				
16	ctg04_114446	PBA HatTrick	0.00638157	Cluster 2	intergenic_region, modifier, EKO05_002463-EKO05_002464	hypothetical protein of unknown function	Non-effectors	LINE/Tad1	No
	ctg04_132375	PBA Seamer	0.00772822	Cluster 2	same as ctg04_114446				

17	ctg04_1366342	PBA HatTrick	0.00638157	Cluster 2	intergenic_region, modifier, EKO05_002887-EKO05_002888	hypothetical proteins of unknown function	EKO05_002887: -3.042 (0.724, Cytoplasmic effector but no signal peptide), EKO05_002888: non-effector	LTR/Gypsy	No
18	ctg05_4856	Genesis090	0.01056547	Cluster 1	downstream_gene_variant, modifier, EKO05_003201	ATP-dependant DNA helicase	non-effector	LTR	No
19	ctg05_298757	PBA Seamer	0.00554413	Cluster 2	intergenic_region, modifier, EKO05_003224-EKO05_003225	hypothetical proteins of unknown function	EKO05_003224: -1.432 (0.504, apoplastic effector), EKO05_003225: -2.315 (0.753 Cytoplasmic effector but no signal peptide)	DNA/TcMar-Fot1	No
20	ctg05_2328653	Genesis090	0.00504442	Cluster 1	intergenic_region, modifier, EKO05_003903-EKO05_003904	EKO05_003903: **hypothetical effector** based on Predector score, EKO05_003904: hypothetical protein of unknown function	EKO05_003903: 2.473 (non-effector), EKO05_003904: non-effector (0.779 Cytoplasmic effector but no signal peptide detected).	LTR	No
	ctg05_2374056	PBA HatTrick	0.00758349	Cluster 1	Same as ctg05_2328653				
		ICC3996	0.00684786	Cluster 1					
21	ctg06_1231	Genesis090	0.04526454	Cluster 2	intergenic_region, modifier, CHR_START-EKO05_003906	ATP-dependant DNA helicase	non-effector	DNA/hAT-Charlie	No
			0.00960261	Cluster 1					
		PBA HatTrick	0.00564256	Cluster 2					

22	ctg06_32631	PBA HatTrick	0.01611868	Cluster 1	intergenic_region, modifier, EKO05_003912-EKO05_003913	EKO05_003912: hypothetical cytoplasmic protein of unknown function with a signal peptide, EKO05_003913: **hypothetical apoplastic effector**	EKO05_003912: 2.473 (non-effector), EKO05_003913: 3.884 (0.89, Apoplastic effector)	DNA/TcMar-Fot1	No
23	ctg06_364193	PBA Seamer	0.00554413	Cluster 2	intergenic_region, modifier, EKO05_003977-EKO05_003978	hypothetical proteins of unknown function	EKO05_003977: -3.338 (0.839, Cytoplasmic effector but no signal peptide), EKO05_003977: non-effector	LTR	No
	ctg06_367615	PBA Seamer	0.00772822	Cluster 2	Same as ctg06_364193				
24	ctg07_33708	PBA Seamer	0.00772822	Cluster 2	intergenic_region, modifier, EKO05_004591-EKO05_004592	hypothetical proteins of unknown function	EKO05_004591: -3.042 (0.587, cytoplasmic effector but no signal peptide), EKO05_004592: -2.076 (0.842, cytoplasmic effector but no signal peptide)	-	No
25	ctg07_619873	PBA HatTrick	0.00638156	Cluster 2	intergenic_region, modifier, EKO05_004769-EKO05_004770	EKO05_004769: Triacylglycerol lipase, EKO05_004770: hypothetical secreted protein with similarity to fungal transcription factors	non-effectors	LTR/Gypsy	No
26	ctg08_4047	ICC3996	0.00502948	Cluster 2	downstream_gene_variant, modifier, EKO05_005191	Hypothetical protein with high similarity to fungal helicases	Non-effector	unknown	No

27	ctg08_98214	PBA Seamer	0.01106706	Cluster 1	intergenic_region, modifier, EKO05_005201-EKO05_005202	EKO05_005201: hypothetical apoplastic effector, EKO05_005202: hypothetical protein of unknown function	EKO05_005201: -1.476 (0.654, Apoplastic effector), EKO05_005202: -3.378 (0.675 Cytoplasmic effector but no signal peptide detected)	LTR/Gypsy	Yes
28	ctg08_448981	PBA Seamer	0.01461423	Cluster 1	intergenic_region, modifier, EKO05_005280-EKO05_005281	EKO05_005280-EKO05: **hypothetical apoplastic effector**, EKO05_005281: hypothetical protein of unknown function	EKO05_005280: -2.911 (0.509, Apoplastic effector), EKO05_005281: non-effector	LTR/Gypsy	Yes
	ctg08_498148	PBA HatTrick	0.00638156	Cluster 2	same as ctg08_448981				
29	ctg08_1002177	PBA Seamer	0.00903923	Cluster 1	intergenic_region, modifier, EKO05_005327-EKO05_005328	EKO05_005327: hypothetical tetrahydrofolate synthase; EKO05_005328: hypothetical protein similar to rapid alkalinization factors (RALFs)	EKO05_005327: non-effector, EKO05_005328: 1.001 (0.739, **Apoplastic effector**)	DNA/TcMar-Fot1	No
		PBA HatTrick	0.00613061	Cluster 1					
	ctg08_1002191	PBA Seamer	0.00903923	Cluster 1	same as ctg08_1002177				
		PBA HatTrick	0.00613061	Cluster 1					
30	ctg08_1071033	PBA HatTrick	0.00638157	Cluster 2	intergenic_region, modifier, EKO05_005332-EKO05_005333	EKO05_005332: hypothetical protein of unknown function, EKO05_005333: hypothetical cytoplasmic protein of unknown function with a signal peptide	EKO05_005332: -2.923 (0.666, Cytoplasmic effector but no signal peptide detected), EKO05_005333: non-effector	DNA/Merlin	No

31	ctg08_1874732	ICC3996	0.0059840	Cluster 2	intergenic_region, modifier, EKO05_005581-CHR_END	hypothetical protein with transmembrane domains and high similarity to sugar transporters in fungi	non-effector	LTR/Gypsy	No
32	ctg09_207905	Genesis090	0.00519918	Cluster3	synonymous_variant, low impact, EKO05_005621	hypothetical protein of unknown function with similarity to fungal glucosyl hydrolases	-1.468 (0.663, cytoplasmic effector but no signal peptide)	-	No
33	ctg09_1023045	Genesis090	0.00638157	Cluster 2	intergenic_region, modifier, EKO05_005894-EKO05_005895	EKO05_005894: hypothetical cytoplasmic protein with similarity to fungal carbohydrate esterases, EKO05_005895: hypothetical protein of unknown function	EKO05_005894: 0.266 (0.704, non-effector), EKO05_005895: non-effector	DNA/TcMar-Fot1	No
34	ctg10_10643	ICC3996<br>ICC3996<br>Genesis090	0.00988308<br>0.00535019<br>0.00754685	Cluster 2<br>Cluster 3<br>Cluster 1	synonymous_variant, low impact, EKO05_006140	hypothetical protein of unknown function	-2.614 (0.522 Cytoplasmic effector but no signal peptide detected)	DNA/Kolobok-H	Yes
35	ctg10_157955	PBA Seamer	0.01461423	Cluster 1	intergenic_region, modifier, EKO05_006148-EKO05_006149	EKO05_006148: hypothetical protein of unknown function, EKO05_006149: hypothetical oxidoreductase	non-effectors	DNA/TcMar-Fot1	Yes
	ctg10_157964	PBA Seamer	0.01461423	Cluster 1	same as ctg10_157955				
	ctg10_157976	PBA Seamer	0.01461424	Cluster 1	same as ctg10_157955				
	ctg10_158053	PBA Seamer	0.01461423	Cluster 1	same as ctg10_157955				
36	ctg10_1065329	PBA Seamer	0.00772822	Cluster 2	synonymous_variant, low impact, EKO05_006457	hypothetical protein of unknown function	Non-effector	-	No

						with similarity to ribosome bioproteinsis protein tsr1			
37	ctg10_1922864	ICC3996	0.00857352	Cluster 2	downstream_gene_variant, modifier, EKO05_006718, EKO05_006719, EKO05_006720	EKO05_006718: hypothetical protein of unknown function, EKO05_006719-EKO05_006720: hypothetical proteins of unknown function with similarity to DNA helicases	EKO05_006718: -3.179 (0.744 cytoplasmic effector but no signal peptide), EKO05_006719: non-effector, EKO05_006720: -3.042 (0.796, cytoplasmic effector but no signal peptide)	DNA/Kolobok-H	No
38	ctg11_4855	Genesis090	0.01338384	Cluster 2	intergenic_region, modifier, CHR_START-EKO05_006721	ATP-dependant DNA helicase	non-effector	DNA/hAT-Charlie	No
39	ctg 11_73427	PBA Seamer	0.00772822	Cluster 2	intergenic_region, modifier, EKO05_006726-EKO05_006727	Hypothetical proteins of unknown function	non-effectors	LTR_retrotransposon	No
40	ctg11_355082	PBA Seamer	0.00554413	Cluster 2	ntergenic_region, modifier, EKO05_006755-EKO05_006756	Hypothetical proteins of unknown function	non-effectors	LTR/Gypsy	No
41	ctg11_500888	PBA Seamer	0.00650038	Cluster 2	intergenic_region, modifier EKO05_006793-EKO05_006794	Hypothetical proteins of unknown function	non-effectors	-	No
42	ctg 11_1696540	PBA Seamer	0.00554413	Cluster 2	intergenic_region, modifier, EKO05_007124-EKO05_007125	EKO05_007124: hypothetical protein with a signal peptide and similarity to peptidases, EKO05_007125: hypothetical protein of unknown function	EKO05_007124: 0.873 (non-effector), EKO05_007125: non-effector	LTR/Gypsy	No

43	ctg12_2872	ICC3996	0.00804649	Cluster 1	upstream_gene_variant, modifier, EKO05_007132, EKO05_007133, EKO05_007134	EKO05_007132: hypothetical protein on unknown function with a transmembrane domain, EKO05_007133: hypothetical DNA helicase, EKO05_007134: hypothetical protein of unknown function	non-effectors	DNA/hAT-Charlie	No
	ctg12_3434	Genesis090	0.005501452	Cluster 2	same as ctg12_2872				
	ctg12_9283	Genesis090	0.00757841	Cluster 1	downstream_gene_variant, modifier, EKO05_007132, EKO05_007133; intergenic_region, modifier, EKO05_007134-EKO05_007135	as listed for ctg12_3434, EKO05_007135: hypothetical protein of unknown function	non-effectors	-	No
	ctg12_10098	ICC3996	0.00821206	Cluster 2	same as ctg12_9283			LINE/Tad1	
		PBA HatTrick	0.00771867	Cluster 1					
	ctg12_64739	PBA Seamer	0.00554413	Cluster 2	same as ctg12_9283			LINE/Tad1	
	ctg12_64766	PBA Seamer	0.00554413	Cluster 2	same as ctg12_9283			LINE/Tad1	
	ctg12_64796	PBA Seamer	0.00554413	Cluster 2	same as ctg12_9283			LINE/Tad1	
44	ctg12_902286	PBA Seamer	0.00554413	Cluster 2	intergenic_region, modifier, EKO05_007365-EKO05_007366	Hypothetical proteins of unknown function	non-effector	LTR/Gypsy	No
45	ctg12_1234964	PBA Seamer	0.01106706	Cluster 1	intergenic_region, modifier, EKO05_007471-EKO05_007472	EKO05_007471: hypothetical protein of unknown function, EKO05_007472: **hypothetical cytoplasmic effector**	EKO05_007471: non-effector, EKO05_007472: -1.071 (0.535, Cytoplasmic effector, signal peptide detected)	LTR/Gypsy	Yes

	ctg12_1234970	PBA Seamer	0.01106706	Cluster 1	same as ctg12_1234964				
	ctg12_1234984	PBA Seamer	0.01106706	Cluster 1	same as ctg12_1234964				
46	ctg12_1732383	PBA HatTrick	0.00638157	Cluster 2	intergenic_region, modifier, EKO05_007599-EKO05_007600	EKO05_007599: **hypothetical apoplastic effector**, EKO05_007600: hypothetical protein of unknown function	EKO05_007599: 1.891 (0.808), Apoplastic effector	LTR/Copia	No
47	ctg13_5628	Genesis090	0.01566084	Cluster 3	missense_variant, moderate impact, EKO05_007602, upstream_gene_variant, modifier, EKO05_007603	Hypothetical protein with a transmembrane domain	non-effector	DNA/Kolobok-H	No
	ctg13_5645	Genesis090	0.00758281<br>0.01533566	Cluster 2<br>Cluster 3	same as ctg13_5628				
	ctg13_6273	ICC3996	0.00671848	Cluster 2	missense_variant, low impact, EKO05_007602	see above	non-effector	DNA/Kolobok-H	No
48	ctg13_39540	PBA Seamer	0.00554413	Cluster 2	intergenic_region, modifier, EKO05_007605-EKO05_007606	Hypothetical proteins of unknown function	non-effectors	-	No
49	ctg13_822157	PBA HatTrick	0.00638157	Cluster 2	intergenic_region, modifier, EKO05_007845-EKO05_007846	Hypothetical proteins of unknown function	non-effectors	LTR/Gypsy	No
50	ctg13_1358890	ICC3996	0.00559696	Cluster 2	intergenic_region, modifier, EKO05_007941-EKO05_007942	EKO05_007941: hypothetical protein with transmembrane domain siliar to fungal oligopeptide transporters, EKO05_007942: hypothetical protein of unknown function	non-effectors	LTR/Gypsy	Yes

51	ctg13_1474245	PBA Seamer	0.00772821	Cluster 2	intergenic_region, modifier, EKO05_007958-EKO05_007959	EKO05_007958: hypothetical glycoside hydrolase, EKO05_007959: hypothetical protein of unknown function	EKO05_007958: -0.94 (0.878, **Apoplastic effector**), EKO05_007959: non-effector	DNA/TcMar-Fot1	Yes
	ctg13_1512039	PBA HatTrick	0.00638157	Cluster 2	same as ctg13_1474245				
52	ctg14_5555	Genesis090	0.00516043	Cluster 2	downstream_gene_variant, modifier, EKO05_007960, upstream_gene_variant, modifier, EKO05_007961	EKO05_007960: hypothetical DNA helicase with a transmembrane domain, EKO05_007961: hypothetical protein of unknown function	non-effectors	DNA/Kolobok-H	No
	ctg14_6054	Genesis090	0.01980639	Cluster 3	missense_variant, moderate impact, EKO05_007960	as listed for ctg14_5555		DNA/Kolobok-H	
			0.02917822	Cluster 2					
		ICC3996	0.02164216	Cluster 2					
	ctg14_7795	PBA Seamer	0.00675340	Cluster 3	missense_variant, moderate impact, EKO05_007960	as listed for ctg14_5555			
	ctg14_7982	Genesis090	0.00624210	Cluster 3	missense_variant, low impact, EKO05_007960	as listed for ctg14_5555			
	ctg14_8013	ICC3996	0.03508566	Cluster 2	missense_variant, moderate impact, EKO05_007960	as listed for ctg14_5555		DNA/Kolobok-H	
53	ctg14_128865	PBA Seamer	0.00772822	Cluster2	upstream_gene_variant, modifier, EKO05_007983, EKO05_007984, EKO05_007985			-	
	ctg14_128972	PBA Seamer	0.00772822	Cluster2	same as ctg14_128865			-	
	ctg14_128985	PBA Seamer	0.00772822	Cluster2	same as ctg14_128865			-	

54	ctg14_1128676	Genesis090	0.0114578	Cluster 1	upstream_gene_variant, modifier, EKO05_008334, EKO05_008335	EKO05_008334: telomere length regulation protein, EKO05_008335: ARP2/3 actin-organizing complex subunit Sop2	non-effectors	-	No
	ctg14_1128681	Genesis090	0.01229865	Cluster 1	same as ctg14_1128676				
55	ctg14_1471881	PBA Seamer<br>Genesis090	0.009731598<br>0.00969599	Cluster 2<br>Cluster 1	intergenic_region, modifier, EKO05_008461-EKO05_008462	EKO05_008461 hypothetical transmembrane protein, putative Mg transporter zinc transport protein, EKO05_008462: **hypothetical apoplastic effector**	EKO05_008461: non effector, EKO05_008462: 0.847 (0.671 - Apoplastic effector)	LTR/Gypsy	Yes
	ctg14_1472550	ICC3996	0.022482043	Cluster 2	same as ctg14_1471881				
	ctg14_1472885	ICC3996	0.007242732	Cluster 3	same as ctg14_1471881				
	ctg14_1473114	PBA HatTrick<br>ICC3996<br>ICC3996	0.00512752<br>0.00732687<br>0.00670026	Cluster 1<br>Cluster 3<br>Cluster 2	same as ctg14_1471881				
	ctg14_1473121	ICC3996<br><br>PBA Seamer<br>PBA HatTrick 	0.01110045<br>0.00791407<br>0.00707533<br>0.00509446<br>0.00512752	Cluster 2<br>Cluster 3<br>Cluster 2<br>Cluster 2<br>Cluster 1	same as ctg14_1471881				
	ctg14_1473129	Genesis090<br>PBA Seamer<br>PBA HAtTrick<br>ICC3996	0.00795573<br>0.005560388<br>0.00512752<br>0.00547121	Cluster 2<br>Cluster 2<br>Cluster 1<br>Cluster 3	same as ctg14_1473121				
	ctg14_1473135	Genesis090	0.01022132	Cluster 2	same as ctg14_1473121				
	ctg14_1474269	Genesis090	0.01648159	Cluster 2	same as ctg14_1473121				

	ctg14_1474335	ICC3996	0.01738445	Cluster 2	same as ctg14_1473121				
	ctg14_1474599	PBA Seamer	0.00561586	Cluster 2	same as ctg14_1473121				
	ctg14_1474618	ICC3996<br>PBA Seamer	0.00883718<br>0.00650151	Cluster 2<br>Cluster 2	same as ctg14_1473121				
	ctg14_1475147	Genesis090	0.005236324	Cluster 2	same as ctg14_1473121				
	ctg14_1486225	PBA HatTrick	0.00638157	Cluster 2	same as ctg14_1473121				
56	ctg14_261308	PBA Seamer	0.00772822	Cluster 2	synonymous_variant, low impact, EKO05_008034	Rho GTPase activating protein	non-effector	-	No
57	ctg14_368054	PBA Seamer	0.00554413	Cluster 2	intergenic_region, modifier, EKO05_008063-EKO05_008064	Hypothetical proteins of unknown function	non-effectors	LTR/Gypsy	No
58	ctg15_4819	Genesis090	0.00614213	Cluster 1	downstream_gene_variant, modifier, EKO05_008466	Hypothetical protein with similarity to DNA helicases	non-effector	LTR_Retrotransposon	No
59	ctg15_958303	Genesis090	0.00519918	Cluster 3	synonymous_variant, low impact, EKO05_008749	Glyceraldehyde 3-phosphate dehydrogenase	0.254	-	No
60	ctg15_1240897	PBA HatTrick	0.00638157	Cluster 2	intergenic_region, modifier, EKO05_008834-EKO05_008835	EKO05_008834: hypothetical protein on unknown function, EKO05_008835: Glycerate 2-kinase	non-effectors	LTR/Gypsy	No
61	ctg15_1498555	PBA HatTrick	0.00638157	Cluster 2	upstream_gene_variant, modifier, EKO05_008903	hypothetical protein of unknown function	non-effector	DNA/Kolobok-H	No
62	ctg15_1503638	Genesis090<br>PBA HatTrick<br>ICC3996<br>ICC3996	0.02617707<br>0.006215497<br>0.005432997<br>0.00520425	Cluster 1<br>Cluster 1<br>Cluster 1<br>Cluster 2	downstream_gene_variant, modifier, EKO05_008905	EKO05_008905: DNA helicase with a transmembrane domain	non-effectors	repeat region, unknown family	
	ctg15_1503666	Genesis090<br>PBA HatTrick<br>ICC3996	0.01738969<br>0.008843174<br>0.006234541	Cluster 1<br>Cluster 1<br>Cluster 1	same as ctg15_1503638				
	ctg15_1503691	Genesis090	0.00613647	Cluster 3	same as ctg15_1503638				

		ICC3996	0.00873476	Cluster 2					
	ctg15_1503696	Genesis090<br>ICC3996	0.00784413<br>0.02923538	Cluster 3<br>Cluster 2	same as ctg15_1503638				
63	ctg16_5960	Genesis090<br><br><br>ICC3996	0.01381917<br>0.00882174<br>0.00882174<br>0.007729018	Cluster 2<br>Cluster 1<br>Cluster 2<br>Cluster 2	upstream_gene_variant, modifier, EKO05_008906, EKO05_008907	EKO05_008906: ATP-dependant helicase with a transmemrane domain, EKO05_008907: hypothetical non-cytoplasmic protein	non-effectors	DNA/Kolobok-H	No
64	ctg16_949437	PBA Seamer	0.00772822	Cluster 2	missense_variant, moderate impact, EKO05_009246	hypothetical protein of unknown function	non-effector	-	No
65	ctg16_1448099	PBA HatTrick	0.00638157	Cluster 2	downstream_gene_variant, modifier, EKO05_009408	hypothetical protein of unknown function	non-effector	DNA/hAT-Charlie	No
66	ctg18_77913	PBA HatTrick	0.00638157	Cluster 2	intergenic_region, modifier, EKO05_009874-EKO05_009875	hypothetical proteins of unknown function	non-effectors	-	No
67	ctg18_543932	PBA Seamer	0.00554413	Cluster 2	intergenic_region, modifier, EKO05_010017-EKO05_010018	hypothetical proteins of unknown function	non-effectors	DNA/TcMar-Fot1	
68	ctg19_438760	PBA Seamer	0.00554413	Cluster 2	intergenic_region, modifier, EKO05_010324-EKO05_010325	EKO05_010324: hypothetical protein of unknown function, EKO05_010325: 3-dehydroquinate dehydratase (3-dehydroquinase)	non-effectors	LTR/Gypsy	No
	ctg19_448773	PBA Seamer	0.00772822	Cluster 2	same as ctg19_438760				
69	ctg19_52221	PBA Seamer	0.00554413	Cluster 2	upstream_gene_variant, modifier, EKO05_010258	Hypothetical **Apoplastic effector**	1.724 (0.711 Apoplastic effector)		
	ctg19_52228	PBA Seamer	0.00554413	Cluster 2	same as ctg19_52221				
70	ctg19_714127	PBA Seamer	0.00554413	Cluster 2	intergenic_region, modifier, EKO05_010378-EKO05_010379	EKO05_010378: hypothetical protein of unknown function,	non-effector	-	No

						EKO05_010379: hypothetical lycerophosphodiester phosphodiesterase			
71	ctg19_1154947	PBA Seamer	0.01089556	Cluster 3	intergenic_region, modifier, EKO05_010492-EKO05_010493	EKO05_010492: **hypothetical apoplastic effector**, EKO05_010493: amino acid transporter with transmembrane domains	EKO05_010492: 2.244 (0.895, Apoplastic effector), EKO05_010493: non-effector	LTR/Gypsy	Yes
		PBA HatTrick	0.005482253	Cluster 3					
	ctg19_1154955	PBA Seamer	0.01089556	Cluster 3	same as ctg19_1154947				
		PBA HatTrick	0.00548225	Cluster 3					
	ctg19_1154999	PBA Seamer	0.01089556	Cluster 3	same as ctg19_1154947				
		PBA HatTrick	0.00504485	Cluster 3					
	ctg19_1155004	PBA Seamer	0.01089556	Cluster 3	same as ctg19_1154947				
		PBA HatTrick	0.005482253	Cluster 3					
	ctg19_1155061	PBA Seamer	0.01089556	Cluster 3	same as ctg19_1154947				
		PBA HatTrick	0.005044855	Cluster 3					
	ctg19_1155087	PBA Seamer	0.01089556	Cluster 3	same as ctg19_1154947				
	ctg19_1155193	PBA Seamer	0.01077375	Cluster 3	same as ctg19_1154947				
	ctg19_1155290	PBA Seamer	0.008570053	Cluster 3	same as ctg19_1154947				
	ctg19_1155317	PBA Seamer	0.01089556	Cluster 3	same as ctg19_1154947				
	ctg19_1155345	PBA Seamer	0.008575561	Cluster 2	same as ctg19_1154947				

72	ctg20_10055	ICC3996	0.01182705	Cluster 3	upstream_gene_variant, modifier, EKO05_010523, EKO05_010524	EKO05_010523: DNA helicase with a transmembrane domain, EKO05_010524: hypothetical non-cytoplasmic protein of unknown function	non-effectors	LTR_retrotransposon	No
		PBA HatTrick	0.005817049	Cluster 1					
	ctg20_6546	ICC3996	0.00554558	Cluster 1	missense_variant, moderate impact, EKO05_010523	as listed for ctg20_10055			
	ctg20_6490	ICC3996	0.01815324	Cluster 2	missense_variant, moderate impact, EKO05_010523	as listed for ctg20_10055			
	ctg20_7536	ICC3996	0.0262593	Cluster 2	same as ctg20_10055				
		Genesis090	0.00555415	Cluster 2					
	ctg20_7609	Genesis090	0.00589039	Cluster 2	same as ctg20_10055				
		ICC3996	0.00621149	Cluster 2					
	ctg20_7659	ICC3996	0.00621149	Cluster 2	same as ctg20_10055				
	ctg20_8917	ICC3996	0.02051209	Cluster 2	same as ctg20_10055				
	ctg20_9902	PBA HatTrick	0.00742752	Cluster 1	same as ctg20_10055				
73	ctg20_254353	Genesis090	0.01066103	Cluster 1	missense_variant, moderate impact, EKO05_010561	hypothetical HECT-type E3 ubiquitin transferase	non-effector	-	No
74	ctg20_332105	ICC3996	0.00949823	Cluster 2	intergenic_region, modifier, EKO05_010582-EKO05_010583	EKO05_010582: **hypothetical apoplastic effector,** EKO05_010583: hypothetical Xylan 1,4-beta-xylosidase with a transmembrane domain	EKO05_010582: 0.981 (0.607, Apoplastic effector), EKO05_010583: non-effector	LTR_retrotransposon	No
	ctg20_332127	ICC3996	0.00949823	Cluster 2	same as ctg20_332105				
	ctg20_342634	PBA Seamer	0.00554413	Cluster 2	same as ctg20_332105				

75	ctg21_153969	PBA HatTrick	0.00638156	Cluster 2	intergenic_region, modifier, EKO05_010892-EKO05_010893	EKO05_010892: acetyltransferase, EKO05_010893: hypothetical protein of unknown function with a transmembrane domain	non-effectors	LTR/Gypsy	No
	ctg21_174491	PBA HatTrick	0.00638156	Cluster 2	same as ctg21_153969				
	ctg21_181970	PBA HatTrick	0.00638157	Cluster 2	same as ctg21_153969				
	ctg21_185186	PBA HatTrick	0.00628248	Cluster 2	same as ctg21_153969				
	ctg22_4536	Genesis090	0.00842176	Cluster 3	downstream_gene_variant, modifier, EKO05_011220, EKO05_011221	EKO05_011220: DNA helicase with a transmembrane domain, EKO05_011221: hypothetical DNA helicase	non-effectors	DNA/Kolobok-H	No
	ctg22_4612	Genesis090	0.00698665	Cluster 1	same as ctg22_4536				
		Genesis090	0.0053572	Cluster 2					
	ctg22_4619	Genesis090	0.00698665	Cluster 1	same as ctg22_4536				
	ctg22_4623	ICC3996	0.00536662	Cluster 1	same as ctg22_4536				
		Genesis090	0.00656978	Cluster 1					
			0.00617011	Cluster 2					

Supp. Table 7. Genetic variations between closely related *Ascochyta rabiei* isolates with contrasting aggressiveness (Pathogenicity Group (PG) 0/1 versus 4/5)

**Pairwise comparison**	**Cluster**	**Variation**	**Variant detected in DAPC**	**DNA region detected in DAPC**	**Variant detected in other pairwise comparisons?**	**Variant type and location**	**Putative protein function**	**Predector Score (EffectorP 3.0 score)**	**Repeat family**	**Region detected in Pyseer**
AR0184 (PG0) and AR0304 (PG4)	2	ctg01_3369118	-	Yes	-	intergenic_region, modifier, EKO05_001005-CHR_END	hypothetical protein of unknown function	non-effector	DNA/Kolobok-H	Yes
		ctg06_1231	Yes	-	Ar0230/AR0226	intergenic_region, modifier, CHR_START-EKO05_003906	ATP-dependant DNA helicase	non-effector	DNA/hAT-Charlie	No
		ctg06_32631	Yes	Yes	AR0037/AR0033, 16RUP013/ andF17191-1	intergenic_region, modifier, EKO05_003912-EKO05_003913	EKO05_003912: hypothetical cytoplasmic protein of unknown function with a signal peptide, EKO05_003913: **hypothetical apoplastic effector**	EKO05_003912: 2.473 (non-effector), EKO05_003913: 3.884 (0.89, Apoplastic effector)	DNA/TcMar-Fot1	No
		ctg13_5645	Yes	Yes	Ar0230/AR0226, AR0219/AR0212, AR0189/AR0179	missense_variant, moderate impact, EKO05_007602	Hypothetical protein with a transmembrane domain	non-effector	DNA/Kolobok-H	No
		ctg14_6054	Yes	-	Ar0230/AR0226, AR0189/AR0179	missense_variant, moderate impact, EKO05_007960	hypothetical DNA helicase with a transmembrane domain	non-effector	DNA/Kolobok-H	No

		ctg14_7982	-	Yes	-	synonymous_variant, low impact, EKO05_007960	hypothetical DNA helicase with a transmembrane domain	non-effector	DNA/Kolobok-H	No
		ctg20_7609	Yes	Yes	AR0189/AR0179	upstream_gene_variant, modifier, EKO05_010523, EKO05_010524	EKO05_010523: DNA helicase with a transmembrane domain, EKO05_010524: hypothetical non-cytoplamic protein of unknown function	non-effectors	LTR_retrotransposon	No
AR0037 (PG0) and AR0033)	2	ctg05_2388561	-	-	-	missense_variant, moderate impact, EKO05_003905	hypothetical DNA helicase with a transmembrane domain	non-effector	DNA/Kolobok-H	No
		ctg06_32631	Yes	Yes	AR0184/AR0304, 16RUP013/F17191-1	See variations between AR0184 (PG0) and AR0304 (PG4)				No
		ctg08_1874732	Yes	Yes	-	intergenic_region, modifier, EKO05_005581-CHR_END	hypothetical protein with transmembrane domains and high similarity to sugar transporters in fungi	non-effector	LTR/Gypsy	No
		ctg10_10643	Yes	Yes	-	synonymous_variant, low impact, EKO05_006140	hypothetical protein of unknown function	-2.614 (0.522 Cytoplasmic effector but no signal peptide detected)	DNA/TcMar-Fot1	Yes
		ctg13_5578	-	Yes	-	synonymous_variant|LOW|EKO05_007602	Hypothetical protein with a transmembrane domain	non-effector	DNA/Kolobok-H	No
		ctg13_6273	-	Yes	-	synonymous_variant|LOW|EKO05_007602-	Hypothetical protein with a transmembrane domain	non-effector	DNA/Kolobok-H	No

16RUP013 (PG1) andF17191-1 (PG5)	1	ctg05_2328653	-	Yes	-	upstream_gene_variant, modifier, EKO05_003903	EKO05_003903: hypothetical effector based on Predector score	EKO05_003903: 2.473 (non-effector)	LTR	No
		ctg05_2328659	-	Yes	-	same as ctg05_2328653				No
		ctg05_2328866	-	Yes	-	same as ctg05_2328653				No
		ctg06_32631	Yes	Yes	AR0184/AR0304, AR0037/AR0033	See variations between AR0184 (PG0) and AR0304 (PG4)				No
		ctg09_10576	-	-	-	stop_gained, high impact, EKO05_005585	hypothetical protein of unknown function	non-effector	DNA/Kolobok-H	No
		ctg14_1128676	Yes	Yes	-	upstream_gene_variant, modifier, EKO05_008334, EKO05_008335	EKO05_008334: telomere length regulation protein, EKO05_008335: ARP2/3 actin-organizing complex subunit Sop2	non-effectors	-	No
		ctg14_1474269	Yes	Yes	-	intergenic_region, modifier, EKO05_008461-EKO05_008462	EKO05_008461 hypothetical transmembrane protein, putative Mg transporter zinc transport protein, EKO05_008462: hypothetical apoplastic effector	EKO05_008461: non effector, EKO05_008462: 0.847 (0.671 - Apoplastic effector)	LTR/Gypsy	Yes
		ctg14_1474599	-	Yes	AR0189/AR0179	same as ctg14_1474269				Yes
		ctg15_1503638	Yes	Yes	-	downstream_gene_variant, modifier, EKO05_008905	EKO05_008905: DNA helicase with a transmembrane domain	non-effectors	repeat region, unknown family	Yes

		ctg15_1503666	Yes	Yes	-	same as ctg15_1503638				Yes
		ctg20_945131	-	-	-	intergenic_region, modifier, EKO05_010755-EKO05_010756	EKO05_010755: hypothetical protein with transmembrane domains and similarity to copper transporters in fungi, EKO05_010756: hypothetical cytoplasmic protein with similarity to alpha-trehalases	EKO05_010755: non-effector, EKO05_010756: 0.37 (non-effector)	LTR/Copia	No
Ar0230 (PG0) and AR0226 (PG4)	3	ctg06_1231	-	Yes	AR0184/AR0304	See variations between AR0184 (PG0) and AR0304 (PG4)				
		ctg13_5391	-	Yes	-	missense_variant, moderate impact, EKO05_007602	Hypothetical protein with a transmembrane domain	non-effector	DNA/Kolobok-H	No
		ctg13_5645	Yes	Yes	AR0184/AR0304, AR0219/AR0212, AR0189/AR0179	missense_variant, moderate impact, EKO05_007602	Hypothetical protein with a transmembrane domain	non-effector	DNA/Kolobok-H	No
		ctg14_6054	Yes	Yes	AR0184/AR0304, AR0189/AR0179	missense_variant, moderate impact, EKO05_007960	EKO05_007960: hypothetical DNA helicase with a transmembrane domain	non-effectors	DNA/Kolobok-H	No
		ctg15_1503691	-	Yes	AR0219/AR0212	downstream_gene_variant, modifier, EKO05_008905	EKO05_008905: DNA helicase with a transmembrane domain	non-effectors	unknown family	Yes
		ctg15_1503696	Yes	Yes	AR0219/AR0212	same as ctg15_1503691				Yes

		ctg17_6257	-	-	-	synonymous_variant, low impact, EKO05_009409|EKO05_009409	hypothetical DNA helicases	non-effector	DNA/Kolobok-H	No
AR0219 (PG0) and AR0212 (PG4)	3	ctg03_2025024	Yes	Yes	-	intergenic_region, modifier, EKO05_002252-EKO05_002253	EKO05_002252-EKO05: hypothetical protein of unknown function, EKO05_002253: hypothetical protein with similarity to oxidoreductases	non-effectors	LTR/Gypsy	Yes
		ctg08_1875607	-	Yes	-	intergenic_region, modifier, EKO05_005581-CHR_END	hypothetical protein with transmembrane domains and high similarity to sugar transporters in fungi	non-effector	LTR/Gypsy	No
		ctg13_5645	Yes	Yes	AR0184/AR0304, Ar0230/AR0226, AR0189/AR0179	missense_variant, moderate impact, EKO05_007602	Hypothetical protein with a transmembrane domain	non-effector	DNA/Kolobok-H	No
		ctg13_870015	-	-	-	intergenic_region, modifier, EKO05_007845-EKO05_007846	EKO05_007845: hypothetical protein of unknown function with transmembrane domains, EKO05_007846: hypothetical protein with similarity to clavaminate synthase proteins in fungi	EKO05_007845: non-effector, EKO05_007846: non-effector	LTR/Gypsy	Yes

ctg14_5555	Yes	Yes	-	downstream_gene_variant, modifier, EKO05_007960, upstream_gene_variant, modifier, EKO05_007961	EKO05_007960: hypothetical DNA helicase with a transmembrane domain, EKO05_007961: hypothetical protein of unknown function	non-effectors	DNA/Kolobok-H	No
ctg14_1527717	-	-	-	missense_variant, moderate impact, EKO05_008465	hypothetical DNA helicase	noneffector	DNA/Kolobok-H	No
ctg14_1527726	-	-	-	upstream_gene_variant, modifier, EKO05_008465	hypothetical DNA helicase	noneffector	DNA/Kolobok-H	No
ctg14_1527758	-	-	-	same as ctg14_1527726				No
ctg15_1503691	-	Yes	Ar0230/AR0226	downstream_gene_variant, modifier, EKO05_008905	EKO05_008905: DNA helicase with a transmembrane domain	non-effectors	unknown family	Yes
ctg15_1503696	Yes	Yes	Ar0230/AR0226	same as ctg15_1503691				Yes
ctg15_1504673	-	Yes	AR0189, AR0179	same as ctg15_1503691				Yes
ctg20_326251	-	Yes	-	intergenic_region, modifier, EKO05_010582-EKO05_010583	EKO05_010582: hypothetical apoplastic effector, EKO05_010583: hypothetical Xylan 1,4-beta-xylosidase with a transmembrane domain	EKO05_010582: 0.981 (0.607, Apoplastic effector), EKO05_010583: non-effector	LTR_retrotransposon	No

		ctg22-12495	-	Yes	-	upstream_gene_variant, modifier, EKO05_011220-EKO05_011221	EKO05_011220: DNA helicase with a transmembrane domain, EKO05_011221: hypothetical DNA helicase	non-effectors	DNA/Kolobok-H	No
AR0189 (PG0) and AR0179 (PG5)	3	ctg01_3363573	Yes	Yes	-	synonymous_variant, low impact, EKO05_001005	hypothetical protein of unknown function	non-effector	DNA/Kolobok-H	No
		ctg13_3621	-	Yes	-	upstream_gene_varian, modifier, EKO05_007603	Hypothetical protein with a transmembrane domain	non-effector	DNA/Kolobok-H	No
		ctg13_5645	Yes	Yes	AR0184/AR0304, Ar0230/AR0226, AR0219/ AR0212	missense_variant, moderate impact, EKO05_007602, upstream_gene_varian, modifier, EKO05_007603	Hypothetical protein with a transmembrane domain	non-effector	DNA/Kolobok-H	No
		ctg14_6054	Yes	Yes	AR0184/AR0304, Ar0230/AR0226	missense_variant, moderate impact, EKO05_007960	EKO05_007960: hypothetical DNA helicase with a transmembrane domain, EKO05_007961: hypothetical protein of unknown function	non-effector	DNA/Kolobok-H	No
		ctg14_1474335	Yes	Yes	-	intergenic_region, modifier, EKO05_008461-EKO05_008462	EKO05_008461 hypothetical transmembrane protein, putative Mg transporter zinc transport protein, EKO05_008462: hypothetical apoplastic effector	EKO05_008461: non effector, EKO05_008462: 0.847 (0.671 - Apoplastic effector)	LTR/Gypsy	No

ctg14_1474599	Yes	Yes	16RUP013/F17191-1	same as ctg14_1474335				
ctg14_1474618	Yes	Yes	-	same as ctg14_1474335				
ctg14_1475238	Yes	Yes	-	same as ctg14_1474335				
ctg15_1504673	-	Yes	AR0219/AR0212	downstream_gene_variant, modifier, EKO05_008905	EKO05_008905: DNA helicase with a transmembrane domain	non-effectors	unknown family	No
ctg20_7609	Yes	Yes	AR0184/AR0304	upstream_gene_variant, modifier, EKO05_010523, EKO05_010524	EKO05_010523: DNA helicase with a transmembrane domain, EKO05_010524: hypothetical non-cytoplamic protein of unknown function	non-effectors	LTR_retrotransposon	No
ctg22_4536	-	Yes	-	downstream_gene_variant, modifier, EKO05_011220, EKO05_011221	EKO05_011220: DNA helicase with a transmembrane domain, EKO05_011221: hypothetical DNA helicase	non-effectors	DNA/Kolobok-H	No
ctg22_10183	-	Yes	-	upstream_gene_variant, modifier, EKO05_011220, EKO05_011221	EKO05_011220: DNA helicase with a transmembrane domain, EKO05_011221: hypothetical DNA helicase	non-effectors	DNA/Kolobok-H	No

Supp. Table 8. Investigation of SNPs associated with recently functionally characterised *Ascochyta rabiei* effector proteins and virulence factors.

Protein	Reference	Protein accession no.	Nucleotide sequence accession no.	Locus tag on ArME14 genome	SNPs no. and annotation
ArPEC25	Singh et al. (2023)	KZM27126.1	JYNV01000078 locus_tag ST47_g1734	EKO05_009010-T1.cds	None
ArF-BAR	Sinha et al. (2021)	KZM20872.1	JYNV01000264 locus_tag ST47_g8005	EKO05_007830-T2.cds	None
ArCRZ1	Simha et al. (2021)	KZM25117.1	JYNV01000138 locus_tag ST47_g3738	EKO05_007256-T1.cds DNA binding transcription factor	ctg12_557620<br>Upstream gene variant, modifier, EKO05_007256-T1<br>Intergenic region, modifier, EKO05_007255-EKO05_007256

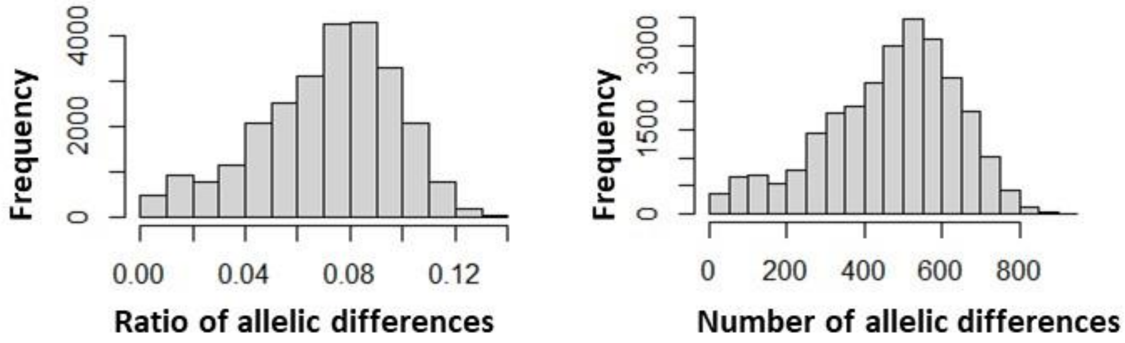

Supp. Fig. 1. Histograms of genetic distances between *Ascochyta rabiei* isolates collected from 2013 to 2020 in Australia, estimated via *diss.dist* function in *poppr*. The histogram on the right shows the number of SNP differences for all pairwise comparisons of *A. rabiei* isolates, and the histogram on the left shows the ratio of the number of observed differences by the number of possible differences.

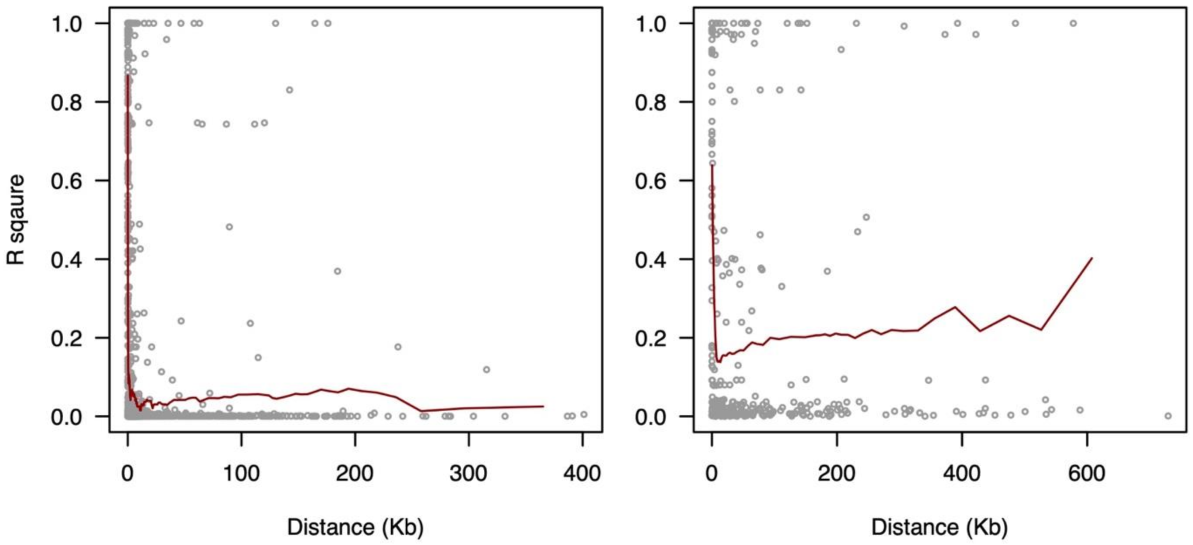

Supp. Fig. 2. Linkage disequilibrium (LD) decay of loci in *Ascochyta rabiei* genome (strain ArME14; NCBI accession GCA_004011695.2) measured as R square of pairwise markers plotted against distance. Linkage disequilibrium was calculated on sliding windows with 100 adjacent genetic markers using GAPIT v.3. for SNP dataset filtered for variants with minimum allele frequency of 1% (left) and 5% (right). The red line is the moving average of the 10 adjacent markers.

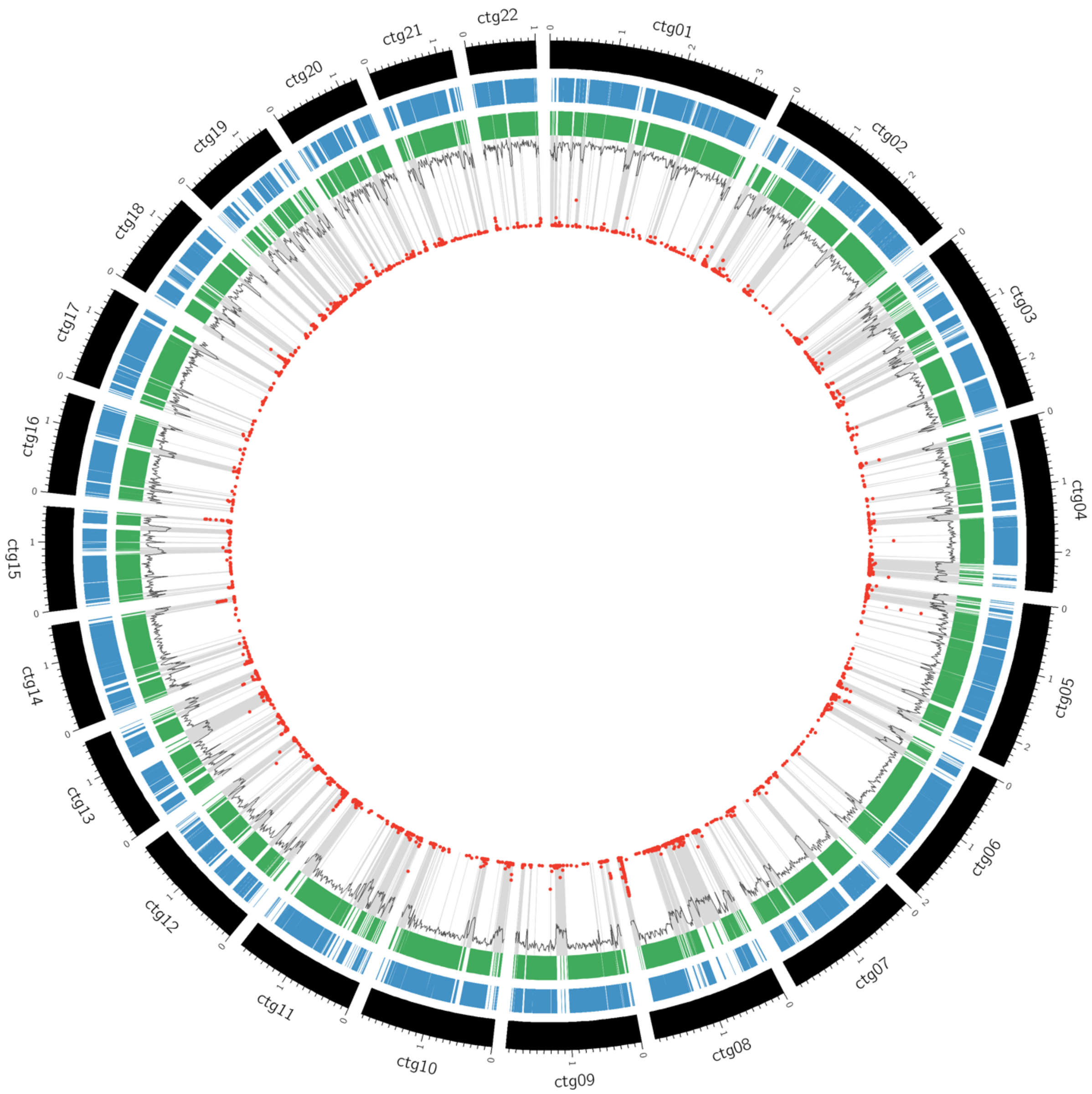

Supp. Fig. 3. CIRCOS plot showing key features of *A. rabiei* ArME14 genome (NCBI accession GCA_004011695.2) and distribution of SNPs, showing *A. rabiei* contigs (black), annotated genes (blue), high GC regions detected in OcculterCut (green), percent GC content (gray histogram). The red points show the frequency of alternate SNP alleles at each site. Highlighted in grey are regions of low GC, which correspond to gene-sparse region and SNP hotspots.

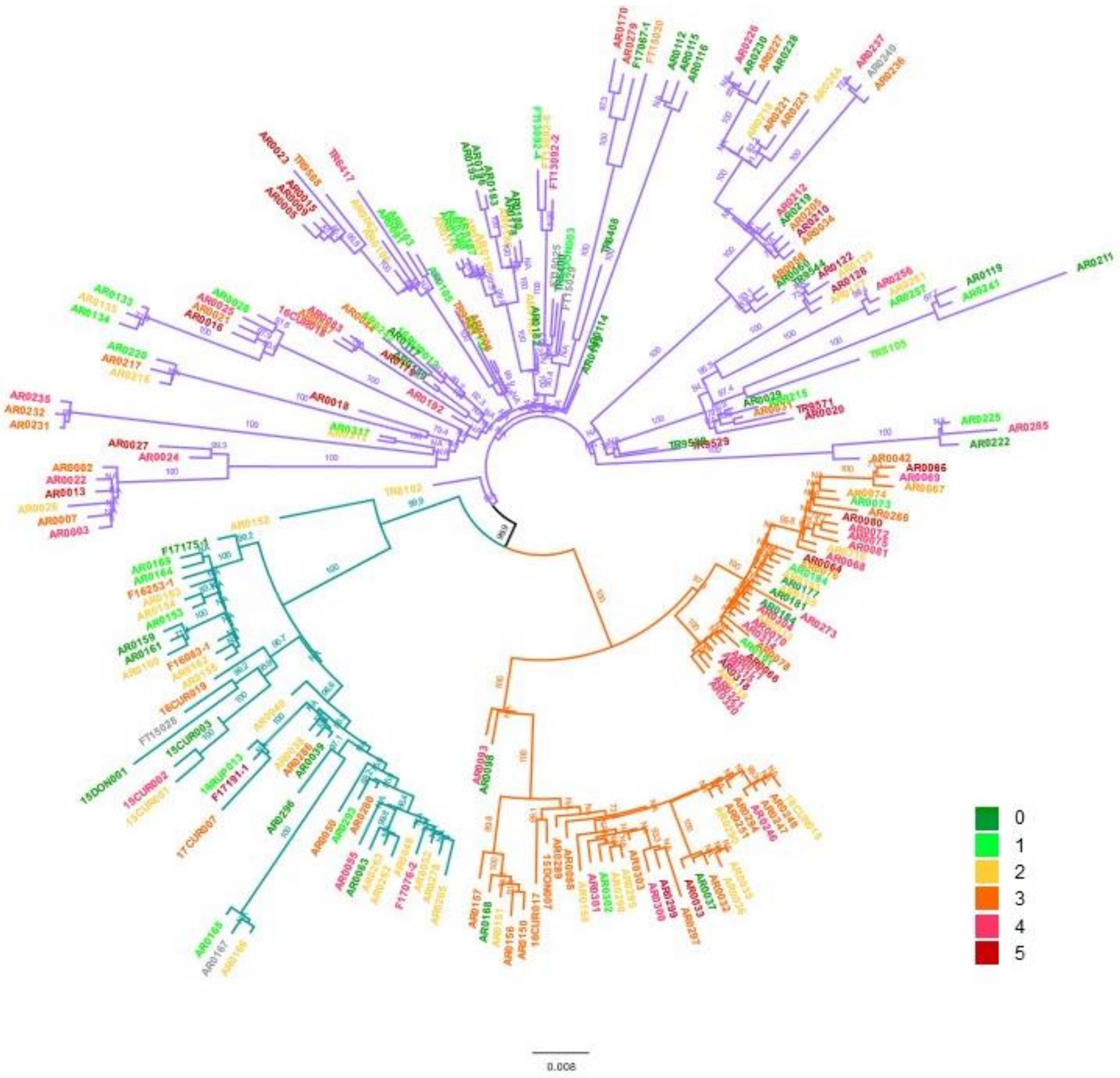

Supp. Fig. 4. Neighbour-joining dendrogram of 230 *Ascochyta rabiei* isolates sequenced in this study based on bitwise genetic distance using 3,283 genome-wide variations. Tip colours correspond to the aggressiveness (pathogenicity group) of the isolates as indicated in the legend. Branches coloured green, orange and purple denote isolates identified in clusters 1, 2, and 3 in the DAPC analysis shown in Figure 1.
